# Agavin induces beneficial microbes in the shrimp microbiota under farming conditions

**DOI:** 10.1038/s41598-022-10442-2

**Published:** 2022-04-16

**Authors:** Juan Pablo Ochoa-Romo, Fernanda Cornejo-Granados, Alonso A. Lopez-Zavala, María Teresa Viana, Filiberto Sánchez, Luigui Gallardo-Becerra, Mirna Luque-Villegas, Yesenia Valdez-López, Rogerio R. Sotelo-Mundo, Andrés Cota-Huízar, Agustín López-Munguia, Adrian Ochoa-Leyva

**Affiliations:** 1 Departamento de Microbiología Molecular, Instituto de Biotecnología (IBT), Universidad Nacional Autónoma de México (UNAM), Av. Universidad #2001, Col. Chamilpa, 62210 Cuernavaca, MOR Mexico; 2 Departamento de Ciencias Químico Biológicas, Universidad de Sonora (UNISON), Blvd., Rosales y Luis Encinas, 83000 Hermosillo, SON Mexico; 3 Instituto de Investigaciones Oceanológicas, Universidad Autónoma de Baja California (UABC), Km 107 carretera Tijuana/Ensenada, 22860 Ensenada, BC Mexico; 4 Laboratorio de Estructura Biomolecular, Centro de Investigación en Alimentación y Desarrollo, A.C. (CIAD), Carretera Gustavo Enrique Astiazarán Rosas Num. 46, Col. La Victoria, 83304 Hermosillo, SON Mexico; 5 Camarones El Renacimiento SPR de RI, Justino Rubio No. 26, Col Ejidal, 81330 Higuera de Zaragoza, SIN Mexico; 6 Departamento de Ingeniería Celular y Biocatálisis, Instituto de Biotecnología, UNAM, Avenida Universidad 2001, Col. Chamilpa, 62420 Cuernavaca, MOR Mexico

**Keywords:** DNA, Microbial communities, Environmental microbiology, Biochemistry, Microbiology, Molecular biology

## Abstract

Prebiotics and probiotics have shown a number of beneficial impacts preventing diseases in cultured shrimps. Complex soluble carbohydrates are considered ideal for fostering microbiota biodiversity by fermentable oligosaccharides, disaccharides, monosaccharides, and polyols (FODMAPS). Here we evaluated the growth performance and microbiota composition of the white shrimp *Litopenaeus vannamei* after dietary intervention using agavin as a FODMAP prebiotic under farming conditions. Adult *L. vannamei* were raised at a shrimp farm and the effect of agavin supplemented at 2% (AG2) or 10% (AG10) levels were compared to an agavin-free basal diet (BD). After 28 days-trial, the feed conversion ratio, total feed ingested, and protein efficiency ratio was significantly improved on animals fed with AG2. At the same time, no effect on growth performance was observed in AG10. Surprisingly, after sequencing the V3–V4 regions of the 16S rRNA gene a higher microbial richness and diversity in the hepatopancreas and intestine was found only in those animals receiving the AG10 diet, while those receiving the AG2 diet had a decreased richness and diversity, both diets compared to the BD. The beta diversity analysis showed a clear significant microbiota clustering by agavin diets only in the hepatopancreas, suggesting that agavin supplementation had a more substantial deterministic effect on the microbiota of hepatopancreas than on the intestine. We analyzed the literature to search beneficial microbes for shrimp’s health and found sequences for 42 species in our 16S data, being significantly increased *Lactobacillus pentosus, Pseudomonas putida* and *Pseudomonas synxantha* in the hepatopancreas of the AG10 and *Rodopseudomonas palustris* and *Streptococcus thermophiles* th1435 in the hepatopancreas of the AG2, both compared to BD. Interestingly, when we analyzed the abundance of 42 beneficial microbes as a single microbial community "meta-community," found an increase in their abundance as agavin concentration increases in the hepatopancreas. In addition, we also sequenced the DNA of agavin and found 9 of the 42 beneficial microbes. From those, *Lactobacillus lactis* and *Lactobacillus delbrueckii* were found in shrimps fed with agavin (both AG2 and AG10), and *Lysinibacillus fusiformis* in AG10 and they were absent the BD diet, suggesting these three species could be introduced with the agavin to the diet. Our work provides evidence that agavin supplementation is associated with an increase of beneficial microbes for the shrimp microbiota at farming conditions. Our study provides the first evidence that a shrimp prebiotic may selectively modify the microbiota in an organ-dependent effect.

## Introduction

The diversity of microorganisms inhabiting the digestive tract of any organism, also known as the microbiota, has a profound effect on the host’s physiology, from nutrient metabolism and immune system development to infection resistance and enhanced growth performance^1–3^. Several studies have shown the importance of the microbiota in their host, even in non-model organisms such as shrimp^4^ The microbiota composition in *L. vannamei* depends on environmental factors such as water salinity and diet, and on biological factors such as the organ and the developmental stage^4–8^.

Shrimp production is the most profitable aquaculture activity globally, mainly due to the rising demand for *L.* *vannamei* and *P. monodon* species^4^. Unfortunately, many factors such as poor water quality, bacterial and viral infections may preclude an optimal shrimp production, yield, and reasonable conversion rate^7,9,10^. In many cases, these complications can be prevented with adequate management in shrimp farming, such as suitable water exchanges and pre and probiotics, considering the restrictions imposed on the antibiotics use^11–14^. Besides, the farm environment strongly influences the microbial interchange between water, sediment, and shrimps^7^. This interchange is essential to maintain an adequate balance between beneficial, innocuous, and pathogenic microbes.

The hepatopancreas and the intestine are critical organs for shrimp nutrition and growth. The hepatopancreas has a fundamental role in energy storage, detoxification, and synthesis of essential hormones, digestive enzymes^15–17^, and crucial molecules for the innate immune system that recognize, bind, and eliminate bacterial pathogens and viruses^18,19^. Additionally, the microbial communities associated with this organ perform functions that help maintain the metabolism and the immune system function^7^. On the other hand, the intestine is mainly involved in nutrient absorption. In general, host health is strongly influenced by the structural integrity of the intestine, the immune molecules, and the microbiota^20^. Many reports dealing with shrimp physiology conclude that, as in many other systems, a healthy intestine microbiota can produce beneficial metabolites such as short-chain fatty acids^21^.

The fishmeal is a traditional ingredient in shrimp diets, but it has become a limiting ingredient due to its high demand^22^, requiring the introduction of an adequate plant protein substitute. Unfortunately, as far as *L. vannamei* is concerned, low fishmeal diets result in impaired growth and a deficient overall performance^23^. Different approaches have modulated shrimp microbiota through diet, improving growth, disease resistance, and productivity. One strategy has been diet supplementation with probiotics, which exert their activity in the shrimp’s digestive tract by inhibiting the proliferation of pathogens, stimulating the immune response, promoting shrimp growth, survival, and nutrient absorption^24,]^^25,]^^26,]^^27^. The second strategy has been through diet supplementation with prebiotics, mainly using polysaccharides such as inulin, amylose, or fructooligosaccharides (FOS), promoting growth, survival, and positive immunological performance, also reducing the relative abundance of potential pathogens^11,28–30^.

More than 15% of higher species contain fructans, which in some species constitute the only reserved carbohydrate. Fructans are also one of the most important nutritional components in human and mammals diet acting both as soluble fiber and prebiotic^31^. Fructans are polymers with linear and branching in β2−1 or β2−6 fructofuranosyl residues, commonly water-soluble and synthesized from sucrose accumulation in the vacuole. Currently a narrow range of confirmed prebiotics exists, with linear chicory inulin dominating the market both as soluble fiber and prebiotic^32^. This contrasts with the fact that prebiotic effects is depend both on the size and structure of fructans^33,34^. Consequently fructan diversity represents an interesting opportunity to design more efficient animal diets. It has been shown that agavin, the fructan component of agave, is a complex mixture of soluble carbohydrates with a wide variety of structural patterns in proportions that depend on the agave species, a combination so unique that led to the proposal to name these inulins as agavin. It has already been shown that as linear inulin, agavin also functions as a dietary fiber, as it is resistant to digestion, promoting beneficial bacteria growth^35–37^. Although agavin is increasingly employed in human diets, its potential as a prebiotic in the culture of marine species has barely been explored. To our knowledge, only four studies have dealt with the role of agavin on shrimps' health, reporting a lower load of white spot syndrome virus (WSSV) and an increased survival^11^, as well as a significant increase in phenoloxidase activity, and in total hemocyte count^38,39^. In addition, agavin acts as a growth promoter, increasing the epithelium's height and the hepatopancreas' tubule area^40^. However, there are no previous reports regarding the agavin effect on the hepatopancreas, and the intestine microbiota of *L. vannamei* analyzed through massive sequencing.

In this study we assessed the agavin effect on the microbiota structure of the intestine and the hepatopancreas, searching in particular for the enrichment of beneficial microbes and validating if the eventual microbiota modifications could be organ-dependent. To explore whether agavin modifies the microbiota in terms of taxonomy, α, and β-diversity profiles under farming conditions, a bioassay inside a pond of a shrimp farm was conducted. After a 28 days trial, we sequenced the V3–V4 hypervariable regions of the 16S rRNA gene and analyzed the microbiota structure in the intestine and hepatopancreas of *L. vannamei*.

## Methods

### Experimental diets

The diets formulation and proximate composition are presented in Table 1. Three isoproteic and isolipidic diets were formulated according to the nutritional requirements for shrimp^41^. The diets contain approximately 375 g kg^−1^ of crude protein and 95 g kg^−1^ of crude lipid with three levels of agavin; the basal diet (BD) without agavin added, the AG2 diet containing 2% w/w of agavin, and AG10 containing 10% w/w of agavin. Corn starch and white wheat flour were used to compensate for the agavin addition.

**Table 1:** Ingredients and proximate composition (g kg^−1^ on a dry matter basis, DM) of diets containing three levels of agavin (0, 2, and 10%) to feed shrimp juveniles (*Litopenaeus vannamei*) under commercial conditions.

Ingredients, g kg^−1^ DM	Agavin levels (g kg^−1^ DM)		
	BD	AG2	AG10
Fishmeal^a^	120	120	120
Poultry byproduct meal^b^	200	200	200
Soybean meal (42% CP)^c^	120	120	120
Corn gluten^d^	40	40	40
White wheat flour	119	61	60
Maizena™ ^e^ (corn starch)	220	257	177
Gelatin^f^	70	70	70
Soybean Oil^g^	48	49	50
Fish oil^h^	30	30	30
Rovimix^i^	10	10	10
Stay C^i^	0.7	0.7	0.7
AGAVIN	0	20	100
Sodium benzoate^j^	2.3	2.3	2.3
BHT^j^	0.1	0.1	0.1
TOTAL	1000	1000	1000
**Proximate composition, g kg**^−1^			
Moisture	26.8	25.8	26.4
Crude Protein	373	375.7	375.5
Crude Lipid	96	95	94
Ash	58	57	55
NFE	446.2	446.5	449.1

NFE (g kg^−1^) = 100% − (crude protein + crude lipid + ash + moisture).

^a^Sardine fishmeal from Proteínas Marinas y Agropecuarias SA de CV, Guadalajara, Jalisco, Mexico.

^b^Pet food grade (65% CP and 14%CF) from Proteínas Marinas y Agropecuarias SA de CV, Guadalajara, Jalisco, Mexico.

^c^Alimentos COLPAC, Hermosillo, Sonora, México.

^d^Ingredión SA de CV, México.

^e^Maizena, Unilever Food Solutions, México.

^f^Progel Mexicana SA de CV, Léon, Guanajuato, México.

^g^Cargill, Minnesota USA.

^h^from sardin (Mazatlán, México).

^i^DSM Nutritional Products México SA de CV, Guadalajara, Jalisco, Mexico, contains in g kg ρ-aminobenzoic acid 1.45; biotin 0.02; myo-inositol 14.5; nicotinic acid 2.9; Capantothenate 1.0; pyridoxine–HCl 0.17; riboflavin 0.73; thiamine-HCl 0.22; menadione 0.17; α-tocopherol 1.45; cyanocobalamine 0.0003; calciferol 0.03; L-ascorbyl-2- phosphate-Mg 0.25; folic acid 0.05; choline chloride 29.65; retinol 0.015; NaCl 1.838; MgSO4·7H2O 6.85; NaH2PO4·2H2O 4.36; KH2PO4 11.99; Ca(H2PO4)2·2H2O 6.79; Fe-citrate 1.48; Ca-lactate 16.35; AlCl3·6H2O 0.009; ZnSO4·7H2O 0.17; CuCl2 0.0005; MnSO4·4H2O 0.04; KI 0.008; CoCl2 0.05 and Stay–C (Vitamin C).

^j^Interquímica SA de CV, Atizapán de Zaragoza, México.

The experimental diets were produced at the facilities of the IIO—UABC, following internal protocols. Briefly, the pulverized ingredients (Inmimex M-300, Mexico) were mixed (Robot Coupe R-60, USA) until obtaining a homogeneous mass. After that, the micronutrients were incorporated into the bulk meal. Next, the oil sources (soybean oil and fish oil) were mixed throughout, and finally, the water containing either, the cooked starch sources and gelatin were added. Diets were then cold-pressed using a commercial-grade (Tor-o-Rey 5HP, Mexico) and dried at 60 °C for 12 h. All feeds were kept cooled (4 °C) throughout the feeding trial.

### Agavin origin and structure

Agavins are inulins, fructose polymers (fructans) obtained for these experiments from the *Agave tequilana Weber var. Azul* as was previously described^42^. Agave fructans used in this study were obtained from 5-year-old *A. tequilana Weber* var. azul plants cultivated in Morelos, Mexico. The plants were donated by the company AGROINDUSTRIA MEXICANA DEL AGAVE MORELENSE A.R. DE I.C. DE R.L, and identical specimens were previously used in another article^42^. No specific permissions were required for the described study and it did not involve endangered or protected species. The Plants were treated according to standard university protocols. For this purpose, the agave fructans were extracted with hot water from 5 kg of shredded pines during 2 h at 70 °C with eventual agitation using an agave:water ratio of 1:1 (w/v). The aqueous extract was centrifuged to eliminate insoluble fibers and spray-dried directly in a Bowen BE-1448 instrument with a nozzle atomizer (Maryland, USA). After that, this substrate was used as the agavin for the diets. Agavins were structurally characterized by gel permeation chromatography (GPC) in a HPLC with a linear Ultrahydrogel column (Waters, Japan) using 0.1 mM NaNO3 at 30 °C as eluent 0.8 mL/min. Using sucrose, 1-ketose, nystose, fructosyl-nystose, as well as dextrans as standards, we determined that these agavins had a weight average molecular weight (Mw) of 5890 Da and a number average molecular weight (Mn) of 3000 Da, with a polydispersity index (PI) of 1.96. This is equivalent to an estimated degree of polymerization of 17 (Fig. S1).

In terms of oligosaccharide content, agavins were characterized by HPAEC-PAD (High Performance Anion Exchange Chromatography coupled to a Pulsed Amperometric Detector) in a Dionex instrument with a CarboPac PA-200 (2 mm × 250 mm) column and an ED40 Electrochemical Detector. The column was equilibrated at 30 °C with 100 mM NaOH (J.T. Baker, Center Valley, U.S.A) at a flow rate of 0.5 mL/min. Fructan oligosaccharides were eluted with a sodium acetate (J.T. Baker, Center Valley, U.S.A) gradient: 5–100 mM in 20 min and 100–300 mM in 40 min, followed by 15 min at 300 mM and 15 min for initial conditions re-equilibration. A wide diversity of fructan oligosaccharides may be observed in the chromatogram obtained in this analysis, demonstrating the complex structure of this fructan source (Fig. S2). This product profile is similar to the one reported for agavins, composed of linear (b2−1) and branched (b2−6) graminans and neofructans^37^.

### Experimental design and management procedure

The bioassay was conducted inside an earth pond (5 ha) of the shrimp farm Camarones el Renacimiento S.P.R. de R.I. in the Northwest Pacific in Sinaloa, Mexico (26° 01′ 55.8″ N 109° 23′ 12.4″ W) during 28 days in the summer of 2016. The bioassay was conducted following the traditional farmer´s rearing conditions which consist in a semi-intensive culture with 20% water exchange/day, feeding twice per day by manual feeding and monitoring food consumption using feeding-trays. It is essential to point out that all biotic and abiotic factors were the same for all specimens during the bioassay since all the cages were submerged in the same pond. 180 apparently healthy shrimps (without signs of rearing stress and physical injuries related to biotic or abiotic factors) of similar weight were randomly assigned into three groups: BD, AG2, and AG10 in three replicates in 9 cages (110 × 110 × 120 cm), submerged inside the pond covered by 90 cm of sea water and containing 20 shrimps per cage (Fig. S3) Shrimps were fed twice daily at 7:00 and 17:00 h using a nylon trays (22 × 22 × 7 cm) with a pore size of 0.1 mm for each cage. The pelletized food was deposited inside the tray, and it was submerged to the bottom of the cage where it remained until the next feeding dose. The initial feeding rate was adjusted to 3% of the total biomass, and it was subsequently adjusted until satiety according to the feeding response of the previous doses. In this manner, the feed doses started supplying the 3% of the initial biomass (~ 600 gr per treatment) daily (divided into two doses), starting with 18 g of food per day 1 per treatment (6 gr per cage). Shrimps were fed twice a day (7:00 and 17:00), the pelleted food was deposited in nylon trays feeders, which were slowly deposited to the bottom of the cage. The pellets were designed to be sinkable as it is widely known that shrimp do not like floating food. The feeder tray was deposited inside each cage and picked up until the next feeding time. If the feeder tray had leftover pellets (< 0.5 gr), the food doses were maintained to the next feeding time. Otherwise, if the feeder tray was empty, the dose was increased for the next feeding time by adding 2 g of food per cage. In the Table S1 shows the used food doses for each treatment per day.

The main water quality variables were monitored twice a day along with temperature and dissolved oxygen using the Ysi pro 20. To analyze the growth performance and feed consumption, all shrimps from each cage were individually weighed at the treatment's initial (day 0) and at the end (day 28).

### Evaluation of growth performance

At the end of the bioassay, the shrimps in each cage were counted and weighted to calculate survival and weight gain (WG), respectively. The weight gain rate (WGR), specific growth rate (SGR), Feed intake (FI), feed conversion ratio (FCR), survival rate (SR), and other growth parameters were calculated according to the following formulas:

Weight gain (WG, g) = final weight − initial weight.

Weight gain rate (WGR, %) = (final weight − initial weight)/initial weight × 100.

Feed conversion rate (FCR) = total feed intake/final weight.

Protein efficiency ratio (PER) = weight gain/total protein intake.

Specific growth rate (SGR, g day^−1^) = ((ln final weight − ln initial weight)/∑ days) × 100.

Thermal growth coefficient (TGC, g day^−1^ °C^−1^) = (final weight^1/3^ − initial weight^1/3^/∑ days × temperature (°C)) × 100.

A one-way ANOVA was performed to detect differences among treatments for statistical analyses.

### Sample collection and DNA sequencing

At the end of the feeding trial, shrimps were starved for 12 h before sampling. To this end, the last feeding dose was at 17:00 of the 28 days-trial, starting the collection of the samples the next morning. This process was made to collect the inhabiting microbiota of the hepatopancreas and intestine, and avoid collecting transitory microbes. In addition, given that we will extract the total DNA to analyze the microbiota, we also want there to be as little DNA as possible from the food. Four shrimps from each cage were randomly selected, and the hepatopancreas and the intestine were aseptically dissected and stored in stabilizer RNA-later solution and stored 72 h at 4 °C and subsequently at − 80 °C until used for microbiota analysis.

According to the manufacturer's recommendations, the total DNA from each organ and diet was extracted using the Quick-DNA Fecal/Soil Microbe Miniprep kit (Zymo research Cat D6010, CA, USA). The DNA integrity and concentration were determined by Agarose gel electrophoresis and Qubit (Invitrogen, Cat. Q33231, CA, USA), respectively. Next, the V3–V4 hypervariable region of the 16S rRNA genes was amplified using the universal primers 338F (5′-ACTCCTACGGGAGGCAGCAG-3') and 533R (5'-TTACCGCGGCTGCTGGCAC-3′), that have been used for studying shrimp microbiota^43^. The PCR reaction system and protocol have been previously described in^8^. The resulting PCR products were purified with Ampure XP beads (Beckman Coulter Inc., Cat. A63881, CA, USA) and barcoded according to the Illumina Sequencing Library Preparation user's guide. Finally, each library's concentration and size distribution was assessed with a Qubit fluorometer and an Agilent 2100 Bioanalyzer (Agilent Technologies, Santa Clara, CA, USA), respectively. The libraries were sequenced in an Illumina MiniSeq platform with a 2 × 250 Paired-End format at the Sequencing Unit from the National Institute of Genomic Medicine, Mexico.

### Bioinformatic analysis

The primers and barcodes were eliminated, and sequences with < Q20 (6 bp sliding window) and containing ambiguous bases were discarded using Trimmomatic. All quality-filtered sequences were joined and analyzed using QIIME (version 1.9). The sequences were clustered into operational taxonomic units (OTUs) based on 97% sequence similarity against the Green Genes (GG) sequence database version 13.8. The singletons were excluded from downstream analyses. The generated OTU table was filtered to discard those OTUs with a total abundance < 0.005%, as previously suggested, to eliminate the very low abundant OTUs^44^. Finally, only the OTUs that met the following criteria were conserved for microbiota analysis: the OTU must appear in more than 50% of the samples of any treatment, and those OTUs that do not meet the above criteria must have a relative frequency equal to or greater than 0.01 (1%), as was previously suggested^8^. Further, OTUs were taxonomically classified using uclust, and the alpha and beta diversity metrics from the OTU table were obtained using QIIME (version 1.9). The alpha diversity metrics were calculated and averaged after 10,000 iterations at a sequencing depth of 4321 reads. The comparison between the alpha diversity indices was evaluated using a Mann–Whitney test (non-parametric test) using a 95% level of confidence (*p* < 0.05). Beta diversity was estimated by computing the weighted and unweighted UniFrac distances among samples from the phylogenetic tree and visualized using a PCoA built with ggplot in R. Finally, the OTUs were subjected to a LefSe analysis to obtain the significantly different OTUs among diets with a significant level (alpha) of 0.05 and the LDA threshold > 2. For all data, statistical significance was set at *p* < 0.05. The significant difference among groups in the distance matrices of every beta analysis was evaluated with ANOSIM.

### Detection of beneficial microbes in the microbiota

We performed a systematic search of all available studies related to shrimp or prawn where beneficial microbes for shrimp health were identified. The SCOPUS database explored the studies using 36 keywords on July 29, 2019 (Table S2). This search resulted in 721 articles from which the title and abstract were screened and selected if they contained experimental results linking the bacteria with a beneficial effect on shrimp health. This process led to 80 bacterial species with positive impact on shrimps’ health (Table S3), resulting from an exhaustive literature search and not user-defined bacteria.

From the 80 beneficial microbes, the Silva132 contained 16S sequences for 70 taxas and Green Genes 13.8 contains for 28 species. Thus, we used the Silva132 database including the 16S sequences for 70 species as a reference list (Table S3) to analyze the presence of the beneficial microbes in our samples. In this manner, the sequence analysis was done considering all species of the ribosomal databases so there is no bias to assign the sequences only to the beneficial bacteria on the list. To this end, we constructed a new BIOM table in which the assignment of OTUs was carried out against Silva132 as a sequence reference, with an identity level of 97%. From the newly generated BIOM table, the taxonomy was assigned at the species level with Qiime 1.9.1, using the command summary_taxa_through_plots.py. The relative abundance for the beneficial microbes was taken from this taxonomy table. Finally, Wilcoxon tests were performed between the abundance of beneficial microbes for each diet and in each organ to determine if a significant enrichment existed in AG2 and AG10 versus BD diets, respectively.

## Results

### Growth performance and nutrient efficiency indices

The temperature (29.96 ± 1.28 °C), dissolved oxygen (4.40 ± 0.50 mg/L), pH (7.69 ± 0.25), and salinity (44.85 ± 0.74 ppt) remained constant and within optimal ranges for culture during the 28-day bioassay (Fig. S4). The shrimps fed with the diet containing 2% agavin (AG2) showed significantly lower FCR (*p* > 0.05) and significantly higher TFI and PER (*p* < 0.05) than BD (Table 2). However, no significant difference was observed in survival and the other evaluated parameters among all the groups (Table 2).

**Table 2:** Growth performance parameters of *L. vannamei*.

Parameters	BD	AG2	AG10	AG2 versus BD	AG10 versus BD
Initial weight (IW) (g)	10.80 ± 0.09	10.90 ± 0.44	11.27 ± 0.20	0.684	0.093
Final weight (FW) (g)	12.87 ± 0.71	13.60 ± 0.42	13.92 ± 0.50	0.158	0.059
Weight gain (WG) (g)	2.07 ± 0.79	2.70 ± 0.19	2.66 ± 0.56	0.22	0.25
Percentage weight gain (PWG) (%)	19.20 ± 7.45	24.81 ± 2.24	23.62 ± 5.25	0.25	0.35
Feed conversion rate (FCR)	2.50 ± 0.50	1.18 ± 0.03	2.64 ± 0.18	0.0019*	0.5823
Total feed intake (TFI) (g)	31.86 ± 5.33	16.10 ± 0.87	36.632 ± 1.10	0.00091*	0.11601
Protein efficiency ratio (PER)	0.19 ± 0.10	0.46 ± 0.04	0.20 ± 0.05	0.0024*	0.8202
Specific growth coefficient (FGC) (g día^−1^)	0.65 ± 0.24	0.82 ± 0.07	0.78 ± 0.16	0.25	0.35
Thermal growth coefficient (TGC) (g día^−1^ °C^−1^)	0.02 ± 0.006	0.021 ± 0.002	0.020 ± 0.004	0.24	0.32
Survival (%)	95 ± 5	100 ± 0	98.33 ± 2.89	0.12	0.27

Values represent means (± S.D.) for three diets with three replicates (n = 9). G2 versus BD and AG10 versus BD columns represent the p-value for each comparison obtained with a Wilcoxon test.All parameters were determined following the formulas mentioned in the methodology.

*Indicates significant *p*-values < 0.05.

Significant values are in bold.

### General microbiota analysis

The DNA from 71 libraries (35 hepatopancreas and 36 intestines) was sequenced with the Illumina Miseq platform, obtaining 2,582,697 sequences after quality filters, with an average of 36,376.01 ± 26,506.94 sequences per sample (Table S4). The Good's coverage revealed we got > 99% of the total OTU’s for intestine and hepatopancreas samples, indicating that the sequencing effort represented the majority of the bacterial communities. Accordingly, the rarefaction curves also suggested an excellent resolution of bacterial communities at the obtained sequencing depth (Fig. S5). After frequency filters (see Methods), we got 724 total OTUs with 97% similarity, from which 97.51% were shared among the three diets (Fig. S6).

### The organ was the main factor influencing the composition of the shrimp microbiota

**Figure 1:**
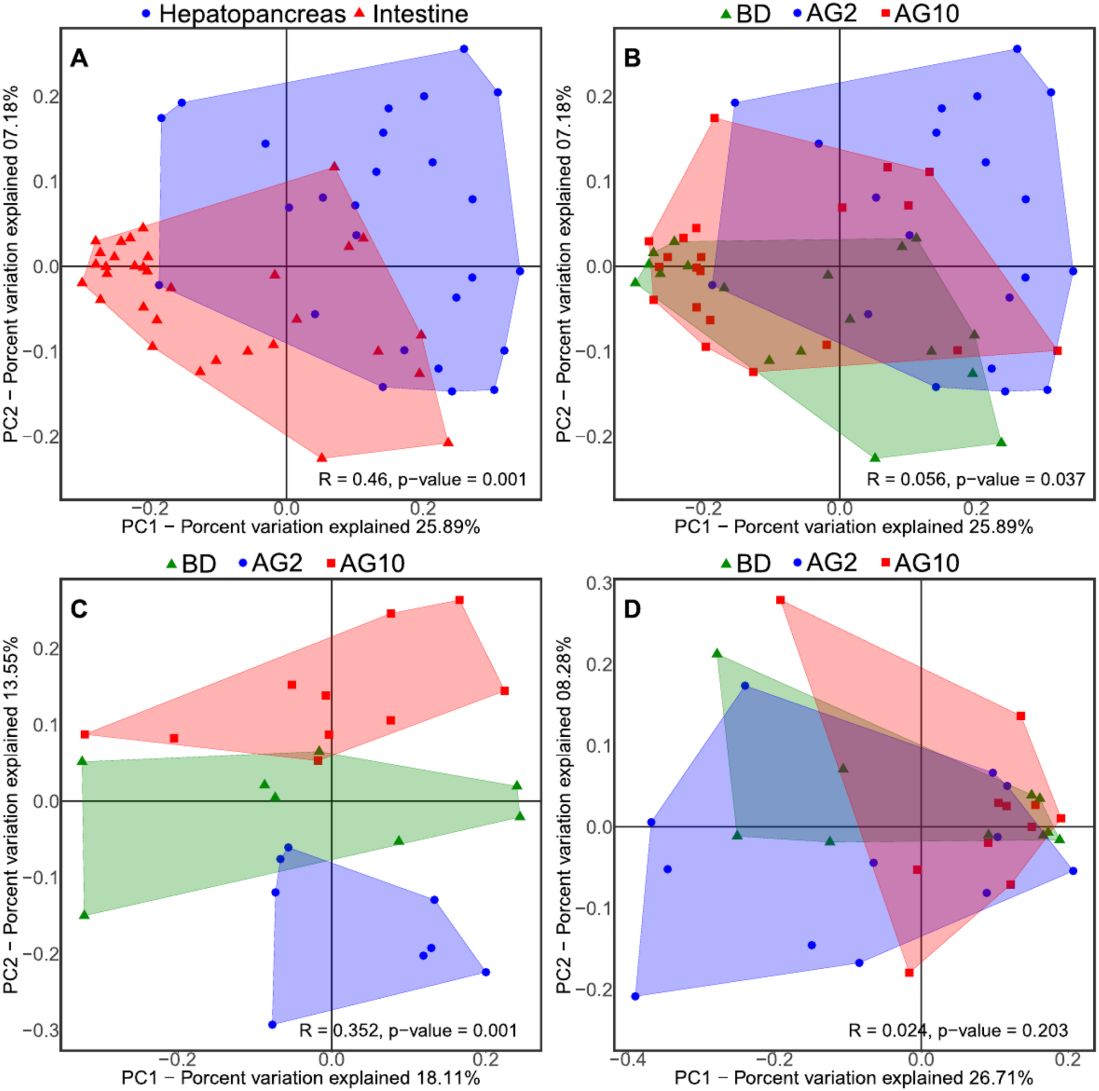
The beta-diversity analysis of microbiota from hepatopancreas and intestine samples. The Unweighted UniFrac distances were used for all PCoA plots. (**A**) Samples tagged by organ. (**B**) Samples tagged by experimental diet. (**C**,**D**) represent the PCoA plots for the microbiota separating the hepatopancreas (**C**) and intestine (**D**), the samples in both plots were tagged by experimental diet.

As a first approach to gain insights into the global differences in the microorganism’s composition, we performed a beta diversity of principal coordinate analysis (PCoA), including all the samples of both organs. The PCoA using UniFrac unweighted distances showed a more precise separation between hepatopancreas and intestine samples (Fig. 1A) than according to the diet (Fig. 1B). Accordingly, the ANOSIM analysis showed that the most critical factor affecting the microbial composition was the organ (R = 0.46, *p* = 0.001), followed by the diet (R = 0.056, *p* = 0.037). The PCoA using UniFrac weighted distances also significantly separated samples according to the organ and diet (Fig. S7). Thus, for further analysis, samples were separated by organ.

### Diet had a more substantial deterministic effect on the microbiota of the hepatopancreas than of the intestine

The beta diversity analyses were carried out separately for each organ to determine the effect of experimental diets. Interestingly, the PCoA using UniFrac unweighted distances showed a clear clustering in the hepatopancreas associated with the diet (Fig. 1C), unlike the intestine, where clusters overlap (Fig. 1D). Similar clustering of samples according to the diet was also observed in the PCoA using UniFrac weighted distances (Fig. S8). The ANOSIM confirmed that the impact of the diet was strong for both Unifrac unweighted (R = 0.352, *p* = 0.001), and weighted distances (R = 0.175, *p* = 0.009) on the hepatopancreas. Contrary, the ANOSIM values for the intestine were non-significant for UniFrac unweighted (R = 0.024, *p* = 0.203), and significant for weighted although with lower values than for diet (R = 0.091, *p* = 0.045). These results suggest that supplementating the diet with agavin has a stronger influence on the hepatopancreas microbiota beta diversity than on the intestine’s.

### Bacterial community was organ-diet dependent

**Figure 2:**
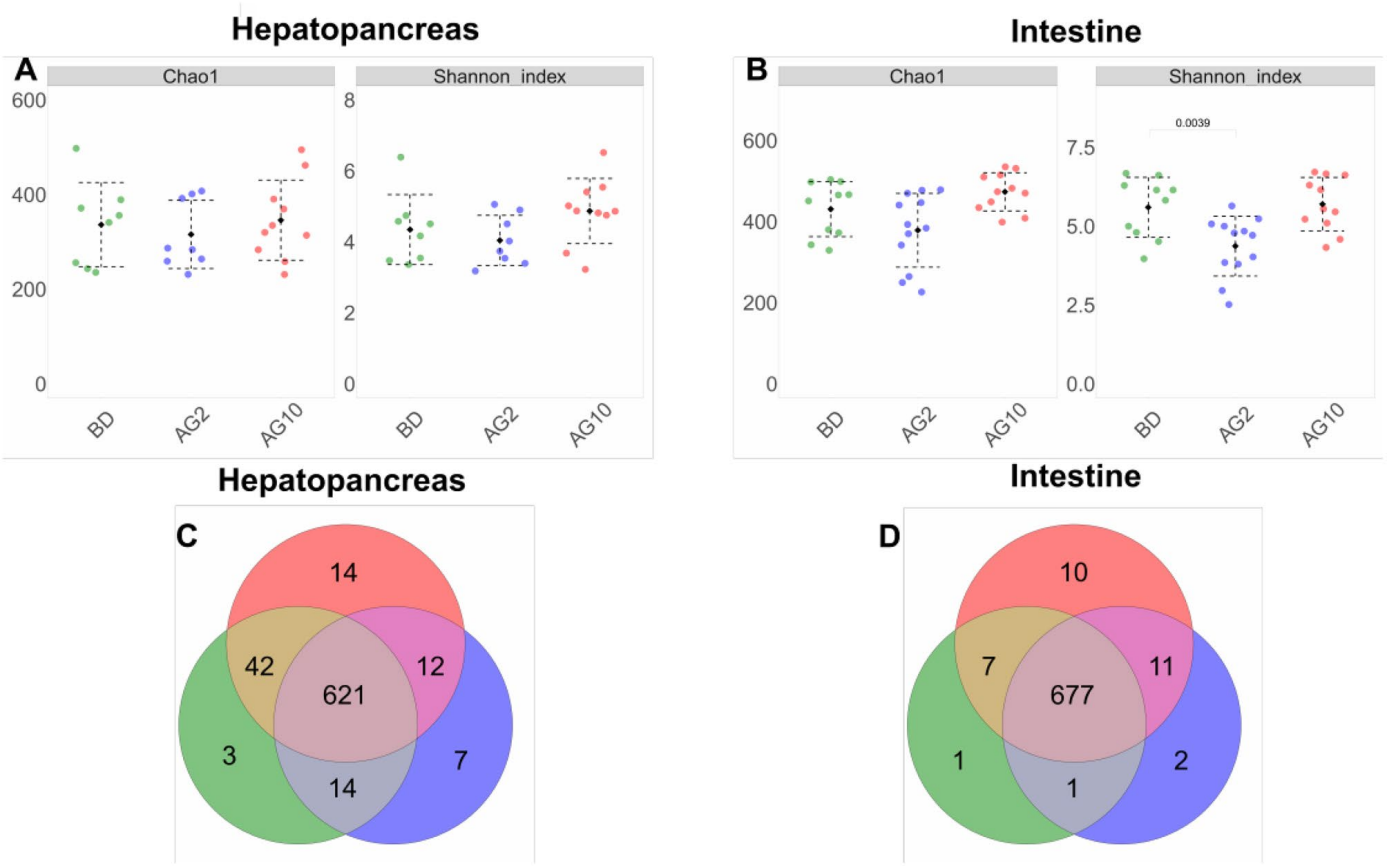
The alpha-diversity analysis and shared OTUs by microbiota. (**A**) Chao1 and Shannon index for the hepatopancreas (**A**) and intestine (**B**) of each treatment. Plots show the mean and standard deviation by each group. (**C**) Venn diagram shows shared and unique OTUs in the hepatopancreas. (**D**) Venn diagram shows shared and unique OTUs in the intestine.

The alpha diversity analysis showed that the AG2 diet seemed to decrease the diversity and richness in both organs, being only significant in the intestine (Fig. 2A,B). Contrary, the AG10 diet tended to increase the alpha diversity and richness in both organs. However, the difference was not significant (Fig. 2A,B).

**Figure 3:**
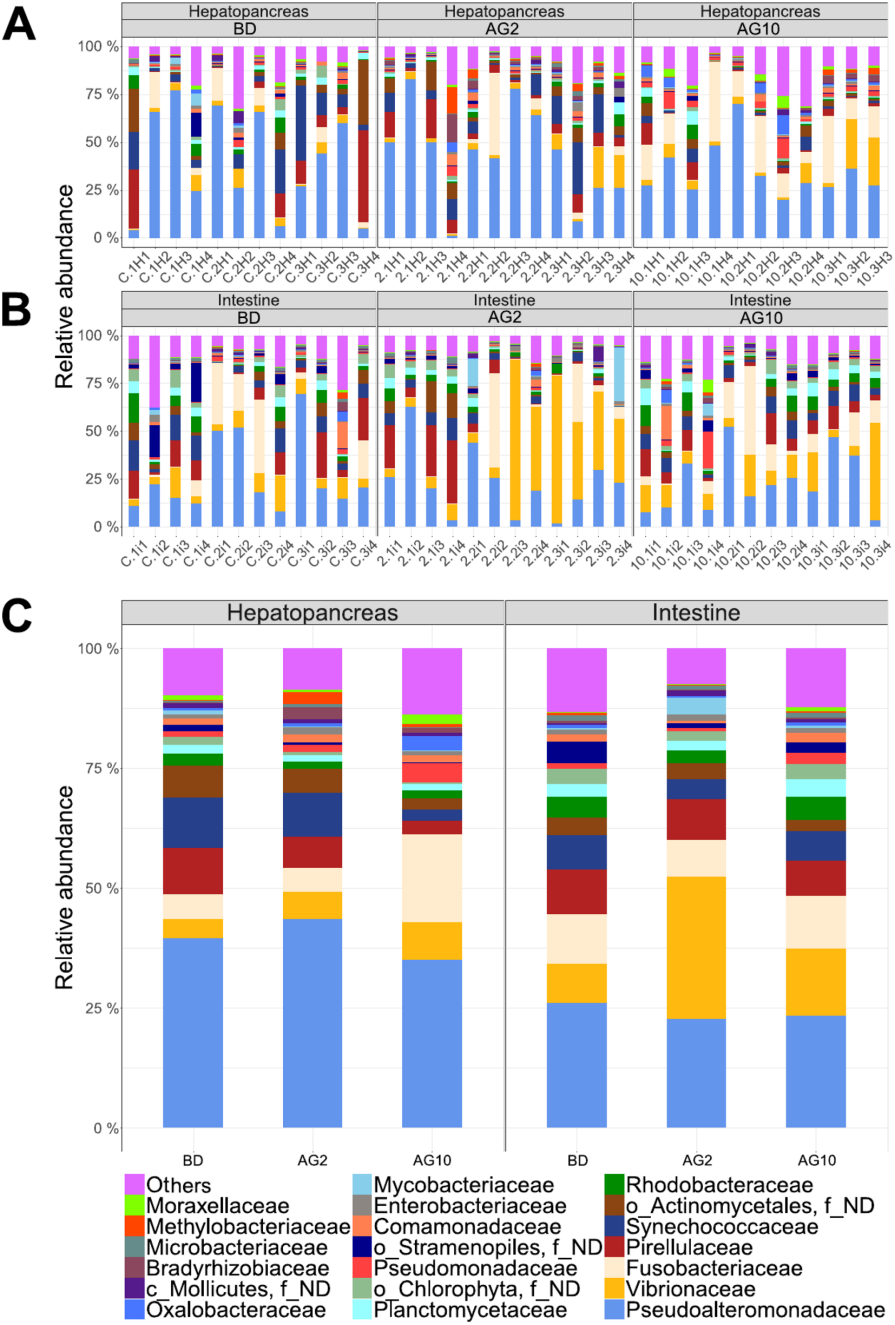
Taxonomic diversity and abundance of all 35 sequenced samples at family level. The Stacked-bar plot represents the relative abundance for (**A**) hepatopancreas and (**B**) intestine samples. (**C**) Relative abundance in the hepatopancreas and intestine for treatment. Only the top 20 is shown; the sum of the remaining taxonomic groups is indicated as “Others”.

We observed that most OTUs were shared among treatments (Fig. 2C,D). The most abundant phyla for hepatopancreas were *Proteobacteria* with 65.31%, 63.17%, and 54.18% for AG2, AG10, and BD diets respectively (Fig. S9) and for intestine with 60.76%, 54.79%, and 49.88% for AG2, AG10 and, BD diets respectively (Fig. S9). In the hepatopancreas, *Pseudoalteromonadaceae* was the most abundant family (Fig. 3), with a higher percentage in AG2 (43.40%), followed by group BD (39.54%) and AG10 (34.90%). In the intestine, *Vibrionaceae* was the most abundant family in AG2 (29.70%), while *Pseudoalteromonadaceae* was the most abundant in BD, and AG10, with 26.04% and 23.34%, respectively (Fig. 3).

**Figure 4:**
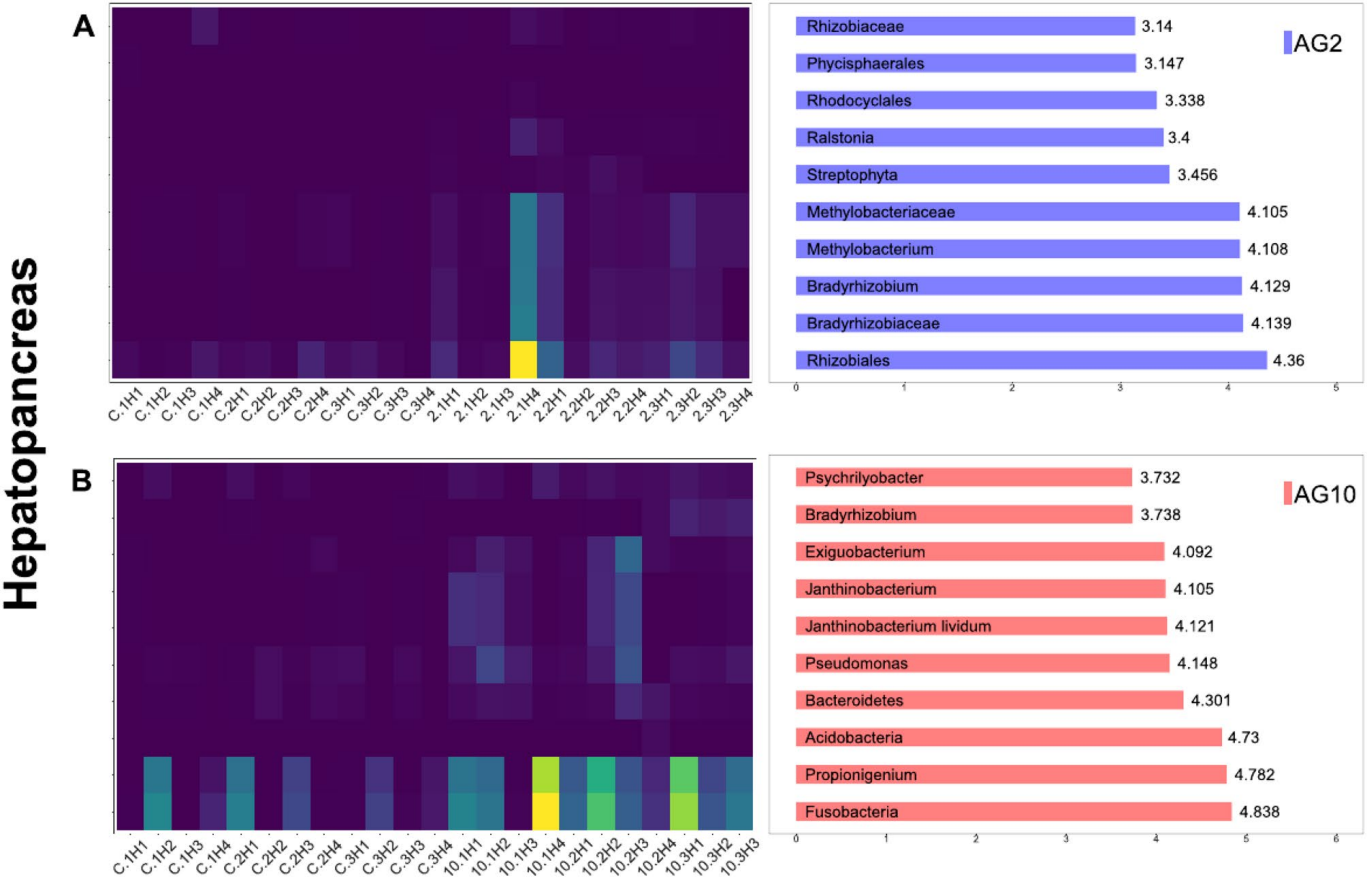
Heatmap of LEfSE results of enriched taxonomic groups in hepatopancreas and their abundance distribution per sample by agavin treatment. (**A**) A2 diet and (**B**) A10 diet. Bar plots represent the log10 LDA score for each classification. Only the top 10 taxonomic levels bearing informative taxonomic labels and LDA effect size > 2(log10) scale are shown. The heatmap shows the abundance of those taxa in the samples with more abundant taxa in yellow.

**Figure 5:**
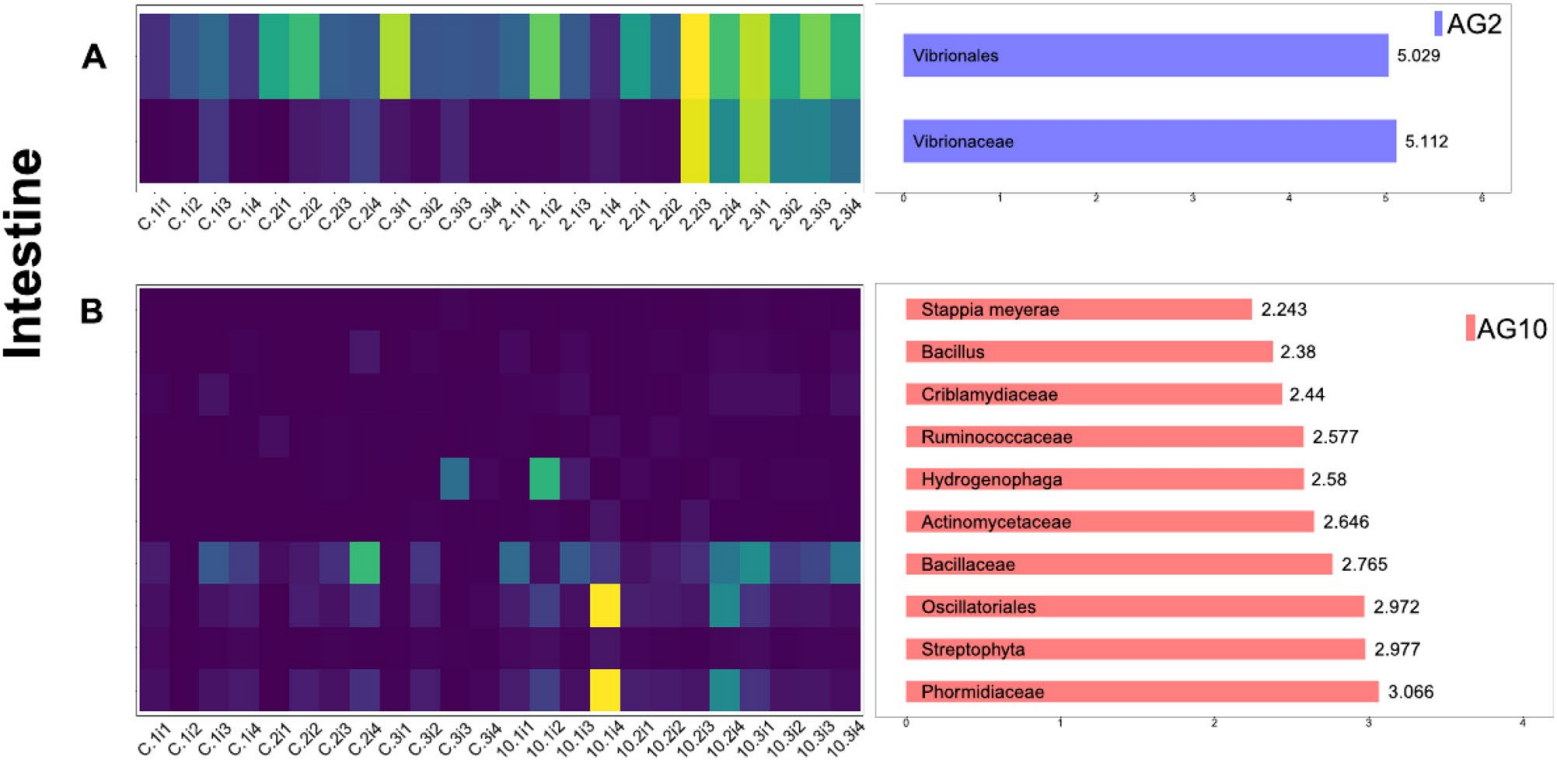
Heatmap of LEfSE results of enriched taxonomic groups in intestines and their abundance distribution per sample by agavin treatment. (**A**) A2 diet and (**B**) A10 diet. Bar plots represent the log10 LDA score for each classification. Only the top 10 taxonomic levels bearing informative taxonomic labels and LDA effect size > 2(log10) scale are shown. The heatmap shows the abundance of those taxa in the samples with more abundant taxa in yellow.

We performed a Lefse analysis for each organ to detect the taxas significantly enriched among diets: in Figs. 4 and 5, the top 10 enriched taxas are listed. When comparing the results obtained with the AG2 versus BD diets in the hepatopancreas, we found 62 significantly enriched taxas in the former. In contrast, when comparing the results obtained with the AG10 versus BD diets, 123 taxas were considerably enriched when the shrimps consumed the AG10 diet. Furthermore, there were fewer differentially enriched taxas in the intestines. When comparing the AG2 versus BD results, we only found eight enriched taxas for the AG2 diet. In contrast, in the AG10 versus BD comparison, 26 significantly enriched taxas were identified when the AG10 diet was consumed. These results suggest that AG10, the diet containing 10% of agavin, induced the enrichment of the highest number of taxas in both organs. The highest impact of diets in differentially abundant taxas in the hepatopancreas was in agreement with the more substantial effect also observed on the beta diversity for this organ.

### Tracking beneficial microbes in hepatopancreas and intestine microbiota

**Figure 6:**
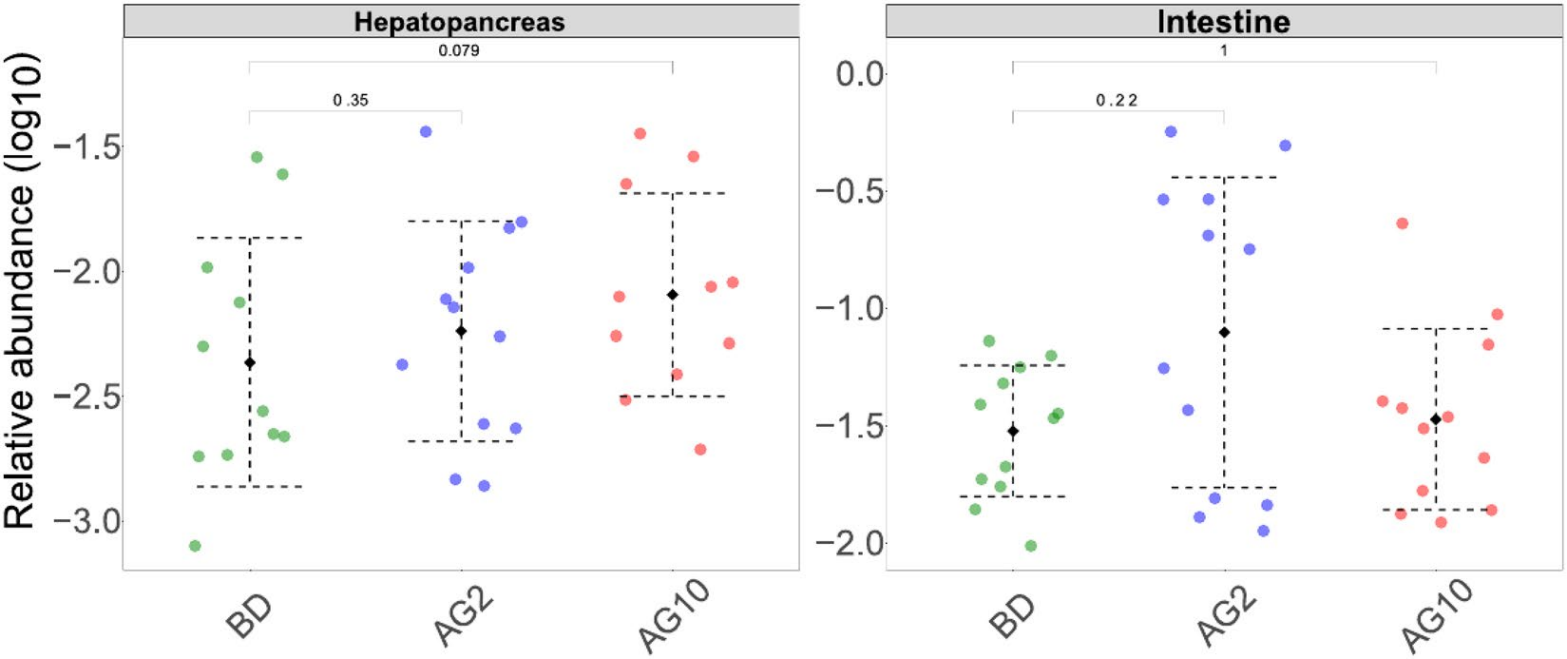
Abundance of beneficial "meta-community" species by each organ and treatment. The boxplots shows the log10 of the sum of the relative abundance frequency of the beneficial microbes for each treatment.

We analyzed the abundance of beneficial microbes in the microbiota of both organs (see Methods). From the 80 species with suggested positive impact on shrimp’s health (Table S3) obtained from the literature search, we found 16S sequences for 42 species in our samples (Table S5). We tested if the abundance of these species increased depending on the diet in each of the organs. We found a significant increased abundance for five species in the hepatopancreas of both agavin diets (Table 3). Interestingly, when we considered the abundance of all beneficial microbes as a single microbial "meta-community," we found an increased abundance in both organs when receiving agavin diets (Fig. 6). However, only the hepatopancreas showed increased meta-community abundance as agavin concentration increased. On the contrary, only the AG2 diet increased the meta-community abundance in the intestine, while the AG10 maintained only a slight increase compared to the control BD diet (Fig. 6).

**Table 3:** Over-abundant probiotic species among diets for hepatopancreas samples.

	BD	AG2	AG10	AG2 versus BD	AG10 versus BD
*Lactobacillus pentosus*	0.000088 ± 0.000209	0.000002 ± 0.000006	0.000588 ± 0.000995	0.51386	0.0087*
*Pseudomonas putida*	0.001694 ± 0.004022	0.001294 ± 0.002322	0.007282 ± 0.010195	0.7723	0.0224*
*Pseudomonas synxantha*	0.000009 ± 0.000030	0.000027 ± 0.000073	0.000105 ± 0.000174	0.18	0.023*
*Rhodopseudomonas palustris*	0.000000 ± 0.000000	0.000043 ± 0.000093	0.000036 ± 0.000112	0.0029*	0.1478
*Streptococcus thermophiles th1435*	0.000000 ± 0.000000	0.000037 ± 0.00074	0.000007 ± 0.000022	0.016*	0.338

AG2 versus BD and AG10 versus BD columns represent the *p*-value obtained with a Wilcoxon test for each comparisson. The values represent means (± S.D). *Indicates significant p-values <0.05. Significant values are in bold.

### Presence of beneficial microbes in the microbiota of Agavin

To identify if the agavin could be the possible origin of the beneficial microbes, we also extracted the total DNA from one agavin sample and sequenced the V3-V4 hypervariable regions, determining the microbiota of the prebiotic.

**Figure 7:**
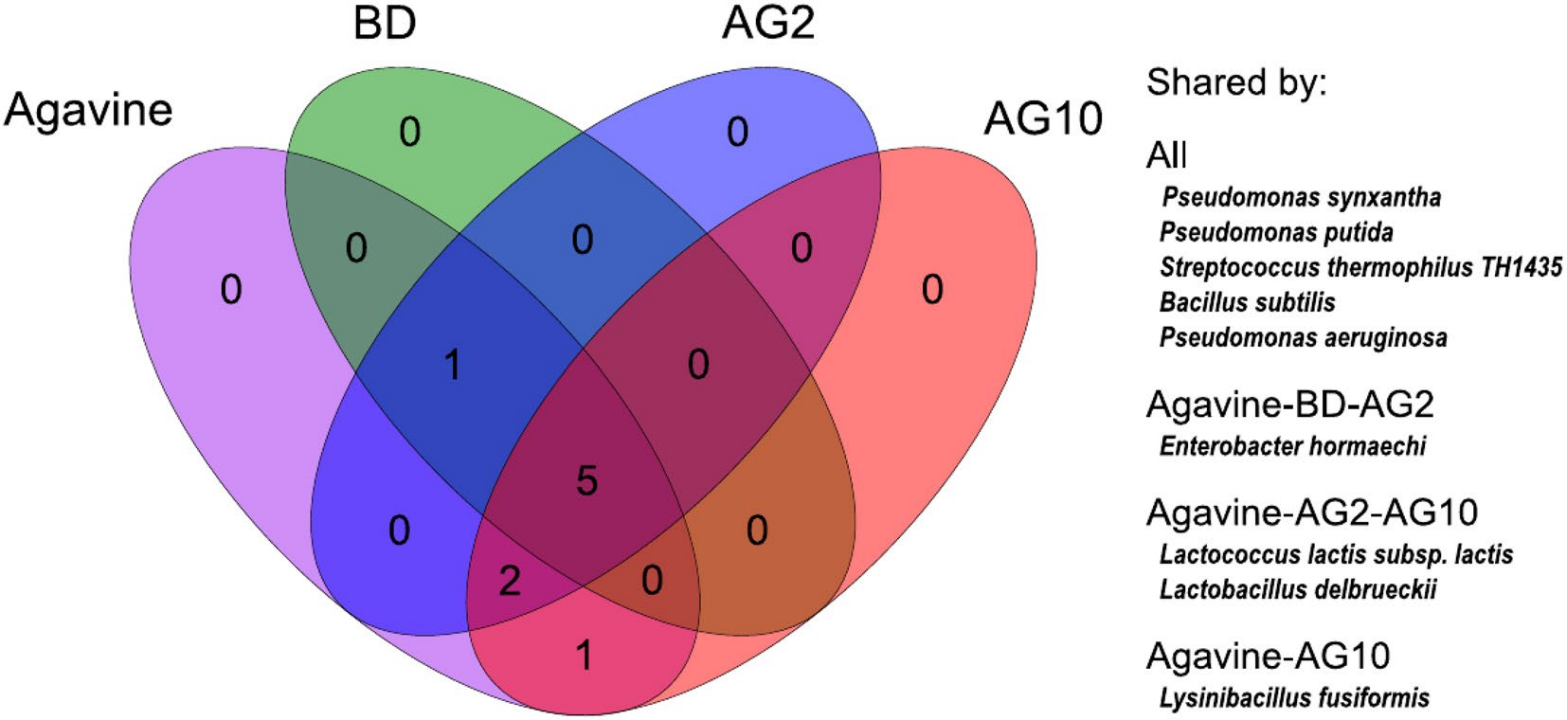
Venn diagram of beneficial microbes found between the agavin and experimental diets.

From the 42 beneficial microbes found in the shrimp microbiota, we observed nine species already present in agavin. Of those nine, five species were present in all shrimp samples (Fig. 7). Interestingly, the other two species (*L. lactis supsp. lactis* and *L. delbrueckii*) were only found in shrimps fed with agavin (both AG2 and AG10) but were absent in shrimps fed with the BD diet (Fig. 7). Furthermore, *L. fusiformis*, present in agavin, was only found in shrimps fed with AG10 (Fig. 7). These results suggest that these three beneficial microbes could have been introduced with agavin, as they were absent in shrimps fed with the BD diet.

## Discussion

It is essential to understand the role the microbiota plays in shrimp health and disease in order to manage its composition during stressful conditions that could affect during production in farms. In this context, several studies have shown the beneficial effect of prebiotics in shrimp production facilities^30,45–49^. In our study, the application of a diet supplemented with 2% agavin showed a favorable impact on the shrimp growth parameters, as revealed by significantly lower FCA and feed intake, in agreement with other studies where dietary fibers are included in the diet as prebiotics^29,45,49,50^. This improvement in the FCA could be related to the significantly higher protein efficiency ratio in the diet obtained with a 2% agavin enriched diet compared to the basal diet, suggesting that changes in the microbiota could be associated with increased protein intake efficiency. In this regard, the same effect was previously observed in the fish *Totoaba macdonaldi* using diets enriched with agavin^51^. Interestingly, a higher proportion of agavin in the shrimp diet (AG10) maintains the same growth performance parameters as the shrimps fed with the BD diet. We suggest that the improvement of growth performance parameters using 2% agavin may be due to a satiety effect that was observed by a lower feed intake, contrary to the observed using 10% agavin. Dose-dependent effects of inulins have been observed in humans, where a higher dose does not necessarily have a better effect, but the opposite may be the case^52^.

Corn starch and white wheat flour were used to compensate for the agavin addition. Both are starch sources, such as cornstarch (pure starch) and white wheat flour (very low protein content), which also contains it even if it comes from different grain (corn and wheat). Both were used to compensate for the amount of agavin in the diets. Not much difference was encountered among treatments considering that figures represent g kg^−1^, being that both together correspond to 33.9, 31.8, and 23.7% in diets. Cooked starch in shrimp did not affect growth or palatability. It is generally used as a filler^53^ to compensate for the ingredients used in a particular experiment. In addition, very large variations in feed component levels such as fibers did not cause any difference in the gut passage time on *L. vannamei*^54^.

The addition of low-dose agavin (2%) to the diet showed a tendency to decrease the microbiota diversity and richness in shrimp hepatopancreas and intestines (Fig. 3), with a reduction in the intestines when compared with the basal diet. The decrease in diversity and richness of the shrimp intestine microbiota has also been observed when alternative prebiotics such as poly-beta-hydroxybutyrate and inulin in the diet has been studied^49,55^. Unexpectedly, a higher dose of agavin (10%) in the diet led to an increased richness and diversity in both organs. However, this difference was not significant when compared with the basal diet. Nevertheless, increases in richness and diversity have also been observed in shrimp intestines using poly-beta-hydroxybutyrate and dietary resistant starch at lower prebiotic concentrations, while diversity and richness decreased at higher prebiotic concentrations^30,55^.

Generally, for different organisms, a higher microbiota diversity is associated with a healthy host condition than a lower diversity one, as a more comprehensive number of species is usually associated with more robust stability, resistance, and resilience to environmental stress, due to functional redundancy^56–58^. However, in humans and mice, an increase in the diversity and richness of the intestine microbiota has been associated with pathological states such as the development of Alzheimer's and metabolic complications related to obesity or aging^59–61^.

Contradictory observations regarding the association of richness and diversity with host health have also been reported for shrimp, where a greater microbial diversity does not necessarily imply a healthier status^62^. For example, the intestines of healthy cultured shrimps have lower diversity and richness than diseased shrimps with AHPND^7^. Nevertheless, it has been also published that AHPND is associated with a significant reduction in bacterial diversity of the stomach compared to that of healthy individuals^63^. In contrast, there is a considerable reduction in the intestine microbial diversity of shrimps with White feces syndrome (WFS)^64^ compared to asymptomatic individuals. Interestingly, there is no change in microbial intestine diversity in shrimps infected with White spot syndrome virus (WSSV) and cotton shrimp-like disease^65,66^.

Our study demonstrates that changes in shrimp microbiota depend on agavin concentration in the diet. The overall microbial richness and diversity and the abundance of probiotic bacteria increased with a high dose of agavin (10%). In contrast, richness, diversity, and the abundance of probiotic bacteria decreased with a low agavin dose (2%). Changes in the shrimp’s intestine microbiota have been observed to be dose-dependent of the prebiotic. For example, low doses (1%) of poly-beta-hydroxybutyrate and resistant starch, induced a high microbial diversity, while higher concentrations (3–5%) reduced the diversity^30,55^. Furthermore, it has also been observed that in diets with a concentration of 0.4% of inulin, the diversity and richness were lower than the control^49^. The effect of prebiotic of decreasing the microbiota richness and diversity may be result from the synthesis of short-chain fatty acids, which have shown a similar effect on the alpha diversity of *L. vannamei*^55^.

The use of prebiotics has been shown to profoundly impact the diversity and richness of the microbiota of *L. vannamei*. Hasyimi et al.^24^ reported that using honey as a prebiotic caused an increase in diversity and richness in the intestines of *L. vannamei* and had a favorable impact on its growth. These results contrast with those reported by Zhou et al.^49^, who observed that inulin causes a decrease in the richness and diversity of the microbiota. The dose-dependent effect of prebiotics has also been observed in other species. The oligosaccharides of bovine origin have been shown to increase the abundance of probiotic bacteria of *Bifidobacterium* while reducing the diversity and richness of the microbiota in infants^67^. Hoffman et al.^68^ reported that mice fed with inulin presented less richness and diversity than mice in the control group. However, inulin-fed mice increased the relative abundance of beneficial taxa such as the genus *Prevotella,* while the abundance of potentially pro-inflammatory genera, such as *Escherichia* and *Turicibacter*, decreased.

It is not easy to control many variables when bioassays are carried out directly under farming conditions. In contrast, the results are closer to technological applications for the aquaculture sector. As it is impossible to observe whether the shrimp are fed under the circumstances of our bioassay, feed consumption was measured from the offered food, and feed adjustments were made daily using the typical doses applied in the shrimp farm. Much of the literature comes from laboratory bioassays where many conditions are controlled, and the results are not transferable to real-life conditions. Our work has precisely addressed the natural environmental variables in semi-intensive field conditions. Recent work addresses the pitfalls and issues of a shrimp bioassay under intensive conditions. In intensive culture systems, environmental variables have been parameterized throughout the culture production tracking protocol. Under commercial production conditions, there is less control of environmental variables than under laboratory conditions or experimental production, so the management protocol seeks to standardize the production pools^50^. The bioassay was designed to evaluate the effect over the microbiota on a 28-days-trial, more than a nutritional study. Whereas the changes in the microbiota have further effects over longer terms is to be addressed in further studies. Studies such as Lee & Lee 2018^69^ reported feeding trials with durations between 36 and 42 days. Another recent work used a 21-days feeding trial to evaluate the effect of probiotics on *L. vannamei* postlarvae and water quality^70^.

The hepatopancreas and intestine of the shrimp are essential organs for nutrient absorption and digestion^62^. Both organs are continuously exposed to external stimuli, including beneficial microbes, pathogens, and many small molecules from food, surrounding sediment and water^62^. Additionally, the hepatopancreas is the main metabolic organ of shrimps^71^. It is the primary source of immune molecules, such as lectins, hemocyanin, ferritin, antibacterial and antiviral proteins, proteolytic enzymes, and nitric oxide^72^. Interestingly, the addition of agavin only exerts a significant effect on the beta-diversity structure of the hepatopancreas, clustering the microbiota according to each diet. In contrast, this effect was not present in the beta-diversity analysis of intestines (Fig. 1). This behavior suggests a deterministic process by adding agavin to the diet, an effect that was strong in the hepatopancreas than intestines.

Furthermore, disease emergence was correlated with reduced deterministic processes that influence microbiota composition and a more stochastic assembly of intestine colonizers^73,74^. Overall, these results suggest that agavin could be an excellent FODMAP prebiotic for hepatopancreas-associated diseases due to the determinist effect in the microbiota structure of this organ. This behavior also indicates that the microbiota structure of the hepatopancreas could be more influenced by prebiotics than the microbiota of the intestine. Interestingly, some studies have revealed that the microbiota of the hepatopancreas has larger stability than the intestine microbiota as it plays a crucial role in host energy and nutrient assimilation^6,75^.

Supplementation with 2% agavin improved growth performance, although the abundance of beneficial microbes was significantly higher at 10% compared to the control. However, the growth performance at 10% was the same observed for the basal diet. This could lead to considering that a food supplemented with 10% agavin offers better protection than 2% against possible pathogens without reducing shrimps' growth performance. These findings clarify that prebiotic concentration is crucial when implementing a prebiotic strategy in shrimp farming.

The use of agavin as a prebiotic capable of promoting a favorable phenotype, and the enrichment of beneficial bacteria genera such as *Lactobacillus* and *Bifidobacterium,* has been widely studied in mice^35,36,76–79^. The intestinal microbiota ferments agavin producing short-chain fatty acids as one of the primary metabolites^77–79^. These are capable of reversing metabolic disorders generated by a high-fat diet in mice^76,77^, and are involved in the optimal development of the epithelium, both of the hepatopancreas and the intestine in shrimp^55^.

The hepatopancreas from AG2 was enriched with genera *Methylobacterium* and *Bradyrhizobium.* Members of *Bradyrhizobium* are capable of nitrogen fixation. Different animals use bacterial nitrogen fixation to compensate for nutritionally unbalanced diets with high carbon and low nitrogen^80,81^. Interestingly, the protein efficiency ratio was significantly higher in the diet with 2% agavin than the control, suggesting that the enriched *Bradyrhizobium* could participate in nitrogen fixation, triggering the increase in protein efficiency ratio. A similar process was recently proposed for *Totoaba macdonaldi*, a predatory fish in which a diet supplemented with 2% agavin also showed higher protein efficiency via enrichment of *Sinorhizobium*, a nitrogen-fixing bacteria genus^51^. On the other hand, the *Bacillaceae* family and specifically the *Bacillus* genus were enriched in the intestines of the AG10 diet. The probiotic potential of *Bacillus* bacteria is well known, a multifunctional probiotic bacterium with a tested capacity to increase aquaculture profitability^82^ There are many articles dealing with the advantage of *Bacillus* as a probiotic in shrimp aquaculture^26,83–88^. More recently, the probiotic effects of *Bacillus* strains directly isolated from shrimp farms have been successfully demonstrated^89^. In this regard, our work suggests that a combination of *Bacillus* species with 10% agavin could be a successful symbiotic for shrimp aquaculture.

Our study clearly shows an agavin dose-dependent influence on the microbiota composition and overall performance of *L. vannamei.* Finally, while the objective of this study was not to elucidate the mechanisms by which the agavin participates in *L. vannamei* metabolism, the inclusion of agavin in the diet, promotes a healthy microbiota via increased abundance of beneficial microbes. Moreover, further research is required to elucidate the role of agavin in improving growth and beneficial microbe’s abundance through research dealing with the expression of genes involved in the immune system, as affected by agavin. To our knowledge this is the first report dealing with the evaluation of diet on the hepatopancreas microbiota composition, suggesting that it is more susceptible to diet than the intestinal microbiota under the conditions employed in our experiments.

## Supplementary Information

Supplementary Information 1.

Supplementary Information 2.

## Data Availability

The data used in this study have been deposited under BioProject PRJNA792615 to the NCBI database.

## References

[1] Wall R, Ross RP, Ryan CA, Hussey S, Murphy B, Fitzgerald GF, Stanton C (2009). Role of intestine microbiota in early infant development. Clin. Med. Pediatr..

[2] Clarke G, Stilling RM, Kennedy PJ, Stanton C, Cryan JF, Dinan TG (2014). Minireview: Intestine microbiota—the neglected endocrine organ. Mol. Endocrinol..

[3] Mueller NT, Bakacs E, Combellick J, Grigoryan Z, Dominguez-Bello MG (2015). The infant microbiome development: Mom matters. Trends Mol. Med..

[4] Cornejo-Granados F, Gallardo-Becerra L, Leonardo-Reza M, Ochoa-Romo JP, Ochoa-Leyva A (2018). A meta-analysis reveals the environmental and host factors shaping the structure and function of the shrimp microbiota. PeerJ.

[5] Rungrassamee W, Klanchui A, Maibunkaew S, Chaiyapechara S, Jiravanichpaisal P, Karoonuthaisiri N (2014). Characterization of intestinal bacteria in wild and domesticated adult black tiger shrimp (Penaeus monodon). PloS one.

[6] Cheung MK, Yip HY, Nong W, Law PTW, Chu KH, Kwan HS, Hui JHL (2015). Rapid change of microbiota diversity in the intestine but not the hepatopancreas during gonadal development of the new shrimp model Neocaridina denticulata. Mar. Biotechnol..

[7] Cornejo-Granados F, Lopez-Zavala AA, Gallardo-Becerra L, Mendoza-Vargas A, Sánchez F, Vichido R, Ochoa-Leyva A (2017). Microbiome of Pacific Whiteleg shrimp reveals differential bacterial community composition between Wild, Aquacultured and AHPND/EMS outbreak conditions. Sci. Rep..

[8] García-López R, Cornejo-Granados F, Lopez-Zavala AA, Sánchez-López F, Cota-Huízar A, Sotelo-Mundo RR, Ochoa-Leyva A (2020). Doing more with less: A comparison of 16S hypervariable regions in search of defining the shrimp microbiota. Microorganisms.

[9] Ferreira NC, Bonetti C, Seiffert WQ (2011). Hydrological and water quality indices as management tools in marine shrimp culture. Aquaculture.

[10] Casillas-Hernández R, Nolasco-Soria H, García-Galano T, Carrillo-Farnes O, Páez-Osuna F (2007). Water quality, chemical fluxes and production in semi-intensive Pacific white shrimp (Litopenaeus vannamei) culture ponds utilizing two different feeding strategies. Aquacult. Eng..

[11] Luna-González A, Almaraz-Salas JC, Fierro-Coronado JA, del Carmen Flores-Miranda M, González-Ocampo HA, Peraza-Gómez V (2012). The prebiotic inulin increases the phenoloxidase activity and reduces the prevalence of WSSV in whiteleg shrimp (Litopenaeus vannamei) cultured under laboratory conditions. Aquaculture.

[12] Luis-Villaseñor IE, Macías-Rodríguez ME, Gómez-Gil B, Ascencio-Valle F, Campa-Córdova ÁI (2011). Beneficial effects of four Bacillus strains on the larval cultivation of Litopenaeus vannamei. Aquaculture.

[13] Le TX, Munekage Y, Kato SI (2005). Antibiotic resistance in bacteria from shrimp farming in mangrove areas. Sci. Total Environ..

[14] Hopkins JS, Hamilton RD, Sandier PA, Browdy CL, Stokes AD (1993). Effect of water exchange rate on production, water quality, effluent characteristics and nitrogen budgets of intensive shrimp ponds. J. World Aquac. Soc..

[15] Van Weel PB (1974). Hepatopancreas?. Comp. Biochem. Physiol. A Physiol..

[16] Yellowlees D (1998). Epithelial cytology and function in the digestive gland of Thenus orientalis (Decapoda: Scyllaridae). J. Crustac. Biol..

[17] Sousa LG, Petriella AM (2000). Histology of the hepatopancreas of the freshwater prawn Palaemonetes argentinus (Crustacea, Caridea). Biocell: Off. J. Soc. Latinoam. de Microsc. Electron..

[18] Cao J, Wang Z, Zhang Y, Qu F, Guo L, Zhong M, Wang X (2014). Identification and characterization of the related immune-enhancing proteins in crab Scylla paramamosain stimulated with rhubarb polysaccharides. Mol. Immunol..

[19] Jiang Q, Zhou Z, Wang L, Wang L, Yue F, Wang J, Song L (2013). A scallop nitric oxide synthase (NOS) with structure similar to neuronal NOS and its involvement in the immune defense. PLoS One.

[20] Levy M, Blacher E, Elinav E (2017). Microbiome, metabolites and host immunity. Curr. Opin. Microbiol..

[21] Koh A, De Vadder F, Kovatcheva-Datchary P, Bäckhed F (2016). From dietary fiber to host physiology: Short-chain fatty acids as key bacterial metabolites. Cell.

[22] Tacon AG, Metian M (2008). Global overview on the use of fish meal and fish oil in industrially compounded aquafeeds: Trends and future prospects. Aquaculture.

[23] Xie S, Liu Y, Tian J, Niu J, Tan B (2020). Low dietary fish meal induced endoplasmic reticulum stress and impaired phospholipids metabolism in juvenile Pacific white shrimp Litopenaeus vannamei. Front. Physiol..

[24] Hasyimi W, Widanarni W, Yuhana M (2020). Growth performance and intestinal microbiota diversity in pacific white shrimp litopenaeus vannamei fed with a probiotic bacterium, honey prebiotic, and synbiotic. Curr. Microbiol..

[25] Javadi A, Khatibi SA (2017). Effect of commercial probiotic (Protexin®) on growth, survival and microbial quality of shrimp (Litopenaeus vannamei). Nutr. Food Sci..

[26] Tepaamorndech S, Chantarasakha K, Kingcha Y, Chaiyapechara S, Phromson M, Sriariyanun M, Visessanguan W (2019). Effects of Bacillus aryabhattai TBRC8450 on vibriosis resistance and immune enhancement in Pacific white shrimp, Litopenaeus vannamei. Fish Shellfish Immunol..

[27] Wang YC, Hu SY, Chiu CS, Liu CH (2019). Multiple-strain probiotics appear to be more effective in improving the growth performance and health status of white shrimp, Litopenaeus vannamei, than single probiotic strains. Fish Shellfish Immunol..

[28] Bolívar Ramírez N, Seiffert WQ, Vieira FDN, Mouriño JLP, Jesus GFA, Ferreira GS, Andreatta ER (2013). Prebiotic, probiotic, and symbiotic-supplemented diet for marine shrimp farming. Pesq. Agrop. Bras..

[29] Hu X, Yang HL, Yan YY, Zhang CX, Ye JD, Lu KL, Sun YZ (2019). Effects of fructooligosaccharide on growth, immunity and intestinal microbiota of shrimp (Litopenaeus vannamei) fed diets with fish meal partially replaced by soybean meal. Aquac. Nutr..

[30] Duan Y, Wang Y, Liu Q, Dong H, Li H, Xiong D, Zhang J (2019). Changes in the intestine microbial, digestion and immunity of Litopenaeus vannamei in response to dietary resistant starch. Sci. Rep..

[31] Kalala G, Kambashi B, Everaert N, Beckers Y, Richel A, Pachikian B, Bindelle J (2018). Characterization of fructans and dietary fibre profiles in raw and steamed vegetables. Int. J. Food Sci. Nutr..

[32] Cunningham M, Azcarate-Peril MA, Barnard A, Benoit V, Grimaldi R, Guyonnet D, Gibson GR (2021). Shaping the future of probiotics and prebiotics. Trends Microbiol..

[33] Wang S, Pan J, Zhang Z, Yan X (2020). Investigation of dietary fructooligosaccharides from different production methods: Interpreting the impact of compositions on probiotic metabolism and growth. J. Funt. Foods.

[34] Astó Sánchez-Lafuente E, Méndez I, Rodríguez-Prado M, Cuñé Castellana J, Espadaler Mazo J, Farran A (2019). Effect of the degree of polymerization of fructans on ex vivo fermented human gut microbiome. Nutrients.

[35] García-Curbelo Y, Bocourt R, Savón LL, García-Vieyra MI, López MG (2015). Prebiotic effect of Agave fourcroydes fructans: An animal model. Food Funct..

[36] Huazano-García A, Shin H, López MG (2017). Modulation of intestine microbiota of overweight mice by agavins and their association with body weight loss. Nutrients.

[37] Mancilla-Margalli NA, López MG (2006). Water-soluble carbohydrates and fructan structure patterns from Agave and Dasylirion species. J. Agric. Food Chem..

[38] Partida-Arangure, B. O., Luna-González, A., Fierro-Coronado, J. A., del Carmen Flores-Miranda, M., & González-Ocampo, H. A. (2013). Effect of inulin and probiotic bacteria on growth, survival, immune response, and prevalence of white spot syndrome virus (WSSV) in Litopenaeus vannamei cultured under laboratory conditions. *Afr. J. Biotechnol*., 12(21).

[39] Intestineiérrez-Dagnino A, Luna-González A, Fierro-Coronado JA, Álvarez-Ruíz P, Flores-Miranda MDC, Miranda-Saucedo S, Escamilla-Montes R (2015). Efecto de la inulina y del ácido fúlvico en la supervivencia, crecimiento, sistema inmune y prevalencia de WSSV en Litopenaeus vannamei. Lat. Am. J. Aquat. Res..

[40] Peña-Rodríguez A, Pelletier-Morreeuw Z, García-Luján J, Rodríguez-Jaramillo MDC, Guzmán-Villanueva L, Escobedo-Fregoso C, Reyes AG (2020). Evaluation of Agave lechuguilla by-product crude extract as a feed additive for juvenile shrimp Litopenaeus vannamei. Aquac. Res..

[41] National Research Council NRC. (2011). Nutrient Requirements of Fish and Shrimp.

[42] Avila-Fernandez A, Rendon Poujol X, Olvera C, Gonzalez F, Capella S, Pena-Alvarez A, Lopez-Munguia A (2009). Enzymatic hydrolysis of fructans in the tequila production process. J. Agric. Food Chem..

[43] Huse SM, Dethlefsen L, Huber JA, Welch DM, Relman DA, Sogin ML (2008). Exploring microbial diversity and taxonomy using SSU rRNA hypervariable tag sequencing. PLoS Genet..

[44] Bokulich NA, Subramanian S, Faith JJ, Gevers D, Gordon JI, Knight R, Caporaso JG (2013). Quality-filtering vastly improves diversity estimates from Illumina amplicon sequencing. Nat. Methods.

[45] Zhou Z, Ding Z, Huiyuan LV (2007). Effects of dietary short-chain fructooligosaccharides on intestinal microflora, survival, and growth performance of juvenile white shrimp, Litopenaeus vannamei. J. World Aquacu. Soc..

[46] Pourmozaffar S, Hajimoradloo A, Miandare HK (2017). Dietary effect of apple cider vinegar and propionic acid on immune related transcriptional responses and growth performance in white shrimp, Litopenaeus vannamei. Fish Shellfish Immunol..

[47] Supungul P, Jaree P, Somboonwiwat K, Junprung W, Proespraiwong P, Mavichak R, Tassanakajon A (2017). A potential application of shrimp antilipopolysaccharide factor in disease control in aquaculture. Aquac. Res..

[48] Li H, Xu C, Zhou L, Dong Y, Su Y, Wang X, Li E (2019). Beneficial effects of dietary β-glucan on growth and health status of Pacific white shrimp Litopenaeus vannamei at low salinity. Fish Shellfish Immunol..

[49] Zhou L, Li H, Qin JG, Wang X, Chen L, Xu C, Li E (2020). Dietary prebiotic inulin benefits on growth performance, antioxidant capacity, immune response and intestinal microbiota in Pacific white shrimp (Litopenaeus vannamei) at low salinity. Aquaculture.

[50] Gainza O, Romero J (2020). Effect of mannan oligosaccharides on the microbiota and productivity parameters of Litopenaeus vannamei shrimp under intensive cultivation in Ecuador. Sci. Rep..

[51] Fuentes-Quesada JP, Cornejo-Granados F, Mata-Sotres JA, Ochoa-Romo JP, Rombenso AN, Guerrero-Rentería Y, Viana MT (2020). Prebiotic agavin in juvenile totoaba, Totoaba macdonaldi diets, to relieve soybean meal-induced enteritis: Growth performance, intestine histology and microbiota. Aquac. Nutr..

[52] Scholz-Ahrens KE, Schaafsma G, van den Heuvel EG, Schrezenmeir J (2001). Effects of prebiotics on mineral metabolism. Am. J. Clin. Nutr..

[53] Tinh TH, Momoh TA, Kokou F, Hai TN, Schrama JW, Verreth JA, Verdegem MC (2021). Effects of carbohydrate addition methods on Pacific white shrimp (Litopenaeus vannamei). Aquaculture.

[54] Beseres JJ, Lawrence AL, Feller RJ (2005). Variation in fiber, protein, and lipid content of shrimp feed: Effects on gut passage times measured in the field. J. Shellfish Res..

[55] Duan Y, Zhang Y, Dong H, Wang Y, Zhang J (2017). Effects of dietary poly-β-hydroxybutyrate (PHB) on microbiota composition and the mTOR signaling pathway in the intestines of litopenaeus vannamei. J. Microbiol..

[56] Naeem S, Li S (1997). Biodiversity enhances ecosystem reliability. Nature.

[57] Le Chatelier E, Nielsen T, Qin J, Prifti E, Hildebrand F, Falony G, Leonard P (2013). Richness of human intestine microbiome correlates with metabolic markers. Nature.

[58] Fan J, Chen L, Mai G, Zhang H, Yang J, Deng D, Ma Y (2019). Dynamics of the intestine microbiota in developmental stages of Litopenaeus vannamei reveal its association with body weight. Sci. Rep..

[59] Hoffman JD, Parikh I, Green SJ, Chlipala G, Mohney RP, Keaton M, Lin AL (2017). Age drives distortion of brain metabolic, vascular and cognitive functions, and the intestine microbiome. Front. Aging Neurosci..

[60] Gallardo-Becerra L, Cornejo-Granados F, García-López R, Valdez-Lara A, Bikel S, Canizales-Quinteros S, Ochoa-Leyva A (2020). Metatranscriptomic analysis to define the Secrebiome, and 16S rRNA profiling of the intestine microbiome in obesity and metabolic syndrome of Mexican children. Microb. Cell Fact..

[61] Bikel S, López-Leal G, Cornejo-Granados F, Gallardo-Becerra L, García-López R, Sánchez F, Ochoa-Leyva A (2021). Intestine dsDNA virome shows diversity and richness alterations associated with childhood obesity and metabolic syndrome. Iscience.

[62] Holt CC, Bass D, Stentiford GD, van der Giezen M (2020). Understanding the role of the shrimp intestine microbiome in health and disease. J. Invertebr. Pathol..

[63] Chen WY, Ng TH, Wu JH, Chen JW, Wang HC (2017). Microbiome dynamics in a shrimp grow-out pond with possible outbreak of acute hepatopancreatic necrosis disease. Sci. Rep..

[64] Hou D, Huang Z, Zeng S, Liu J, Wei D, Deng X, He J (2018). Intestinal bacterial signatures of white feces syndrome in shrimp. Appl. Microbiol. Biotechnol..

[65] Wang J, Huang Y, Xu K, Zhang X, Sun H, Fan L, Yan M (2019). White spot syndrome virus (WSSV) infection impacts intestinal microbiota composition and function in Litopenaeus vannamei. Fish Shellfish Immunol..

[66] Zhou L, Chen C, Xie J, Xu C, Zhao Q, Qin JG, Li E (2019). Intestinal bacterial signatures of the “cotton shrimp-like” disease explain the change of growth performance and immune responses in Pacific white shrimp (Litopenaeus vannamei). Fish Shellfish Immunol..

[67] Simeoni U, Berger B, Junick J, Blaut M, Pecquet S, Rezzonico E, Szajewska H (2016). Intestine microbiota analysis reveals a marked shift to bifidobacteria by a starter infant formula containing a synbiotic of bovine milk-derived oligosaccharides and B ifidobacterium animalis subsp. lactis CNCM I-3446. Environ. Microbiol..

[68] Hoffman JD, Yanckello LM, Chlipala G, Hammond TC, McCulloch SD, Parikh I, Lin AL (2019). Dietary inulin alters the intestine microbiome, enhances systemic metabolism and reduces neuroinflammation in an APOE4 mouse model. PloS one.

[69] Lee C, Lee KJ (2018). Dietary protein requirement of Pacific white shrimp Litopenaeus vannamei in three different growth stages. Fish. Aquat. Sci..

[70] Cai Y, Yuan W, Wang S, Guo W, Li A, Wu Y, Zhou Y (2019). In vitro screening of putative probiotics and their dual beneficial effects: to white shrimp (Litopenaeus vannamei) postlarvae and to the rearing water. Aquaculture.

[71] Vogt G (2019). Functional cytology of the hepatopancreas of decapod crustaceans. J. Morphol..

[72] Rőszer T (2014). The invertebrate midintestinal gland (“hepatopancreas”) is an evolutionary forerunner in the integration of immunity and metabolism. Cell Tissue Res..

[73] Zhu J, Dai W, Qiu Q, Dong C, Zhang J, Xiong J (2016). Contrasting ecological processes and functional compositions between intestinal bacterial community in healthy and diseased shrimp. Microb. Ecol..

[74] Xiong J, Zhu J, Dai W, Dong C, Qiu Q, Li C (2017). Integrating intestine microbiota immaturity and disease-discriminatory taxa to diagnose the initiation and severity of shrimp disease. Environ. Microbiol..

[75] Tzuc JT, Escalante DR, Herrera RR, Cortés GG, Ortiz MLA (2014). Microbiota from Litopenaeus vannamei: Digestive tract microbial community of Pacific white shrimp (Litopenaeus vannamei). Springerplus.

[76] Santiago-García PA, López MG (2014). Agavins from Agave angustifolia and Agave potatorum affect food intake, body weight gain and satiety-related hormones (GLP-1 and ghrelin) in mice. Food Funct..

[77] Huazano-García A, López MG (2015). Agavins reverse the metabolic disorders in overweight mice through the increment of short chain fatty acids and hormones. Food Funct..

[78] González-Herrera SM, Rocha-Guzmán NE, Simental-Mendía LE, Rodríguez-Herrera R, Aguilar CN, Rutiaga-Quiñones OM, Gamboa-Gómez CI (2019). Dehydrated apple-based snack supplemented with Agave fructans exerts prebiotic effect regulating the production of short-chain fatty acid in mice. J. Food Process. Preserv..

[79] González-Herrera SM, Simental-Mendía LE, López MG, Rocha-Guzmán NE, Rutiaga-Quiñones OM, Rodríguez-Herrera R, Gamboa-Gómez CI (2019). Effect of agave fructans on the production of short chain fatty acid in mice. Food Sci. Biotechnol..

[80] Igai K, Itakura M, Nishijima S, Tsurumaru H, Suda W, Tsutaya T, Umezaki M (2016). Nitrogen fixation and nifH diversity in human intestine microbiota. Sci. Rep..

[81] Montes-Grajales, D., Jiménez, B., Rogel, M. A., Alagón, A., Esturau-Escofet, N., Esquivel, B., & Martínez-Romero, E. (2019). Nitrogen-fixing Klebsiella variicola in feces from herbivorous tortoises. bioRxiv, 666818.

[82] Olmos J, Acosta M, Mendoza G, Pitones V (2020). Bacillus subtilis, an ideal probiotic bacterium to shrimp and fish aquaculture that increase feed digestibility, prevent microbial diseases, and avoid water pollution. Arch. Microbiol..

[83] Hindu SV, Chandrasekaran N, Mukherjee A, Thomas J (2017). Effect of dietary supplementation of novel probiotic bacteria Bacillus vireti 01 on antioxidant defence system of freshwater prawn challenged with Pseudomonas aeruginosa. Probiotics Antimicrob. Proteins.

[84] Hindu SV, Thanigaivel S, Vijayakumar S, Chandrasekaran N, Mukherjee A, Thomas J (2018). Effect of microencapsulated probiotic Bacillus vireti 01-polysaccharide extract of Gracilaria folifera with alginate-chitosan on immunity, antioxidant activity and disease resistance of Macrobrachium rosenbergii against Aeromonas hydrophila infection. Fish Shellfish Immunol..

[85] Purivirojkul W, Maketon M, Areechon N (2005). Probiotic properties of bacillus pumilus, bacillus sphaericus and bacillus subtilis in black tiger shrimp (penaeus monodon fabricius) culture. Agric. Nat. Resour..

[86] Nimrat S, Suksawat S, Boonthai T, Vuthiphandchai V (2012). Potential Bacillus probiotics enhance bacterial numbers, water quality and growth during early development of white shrimp (Litopenaeus vannamei). Vet. Microbiol..

[87] Li K, Zheng T, Tian Y, Xi F, Yuan J, Zhang G, Hong H (2007). Beneficial effects of Bacillus licheniformis on the intestinal microflora and immunity of the white shrimp Litopenaeus vannamei. Biotechnol. Lett..

[88] Hao K, Liu JY, Ling F, Liu XL, Lu L, Xia L, Wang GX (2014). Effects of dietary administration of Shewanella haliotis D4, Bacillus cereus D7 and Aeromonas bivalvium D15, single or combined, on the growth, innate immunity and disease resistance of shrimp, Litopenaeus vannamei. Aquaculture.

[89] Kewcharoen W, Srisapoome P (2019). Probiotic effects of Bacillus spp. from Pacific white shrimp (Litopenaeus vannamei) on water quality and shrimp growth, immune responses, and resistance to Vibrio parahaemolyticus (AHPND strains). Fish Shellfish Immunol..

