# Supplementary material for: Agavin induces beneficial microbes in the shrimp microbiota under farming conditions
Source: Sci Rep. 2022 Apr 16;12:6392. doi: 10.1038/s41598-022-10442-2 (PMC9013378; doi:10.1038/s41598-022-10442-2)
Supplement: Supplementary file 1 — Supplementary Information 1. [file 41598_2022_10442_MOESM1_ESM.zip › fig_new_S4.pdf]

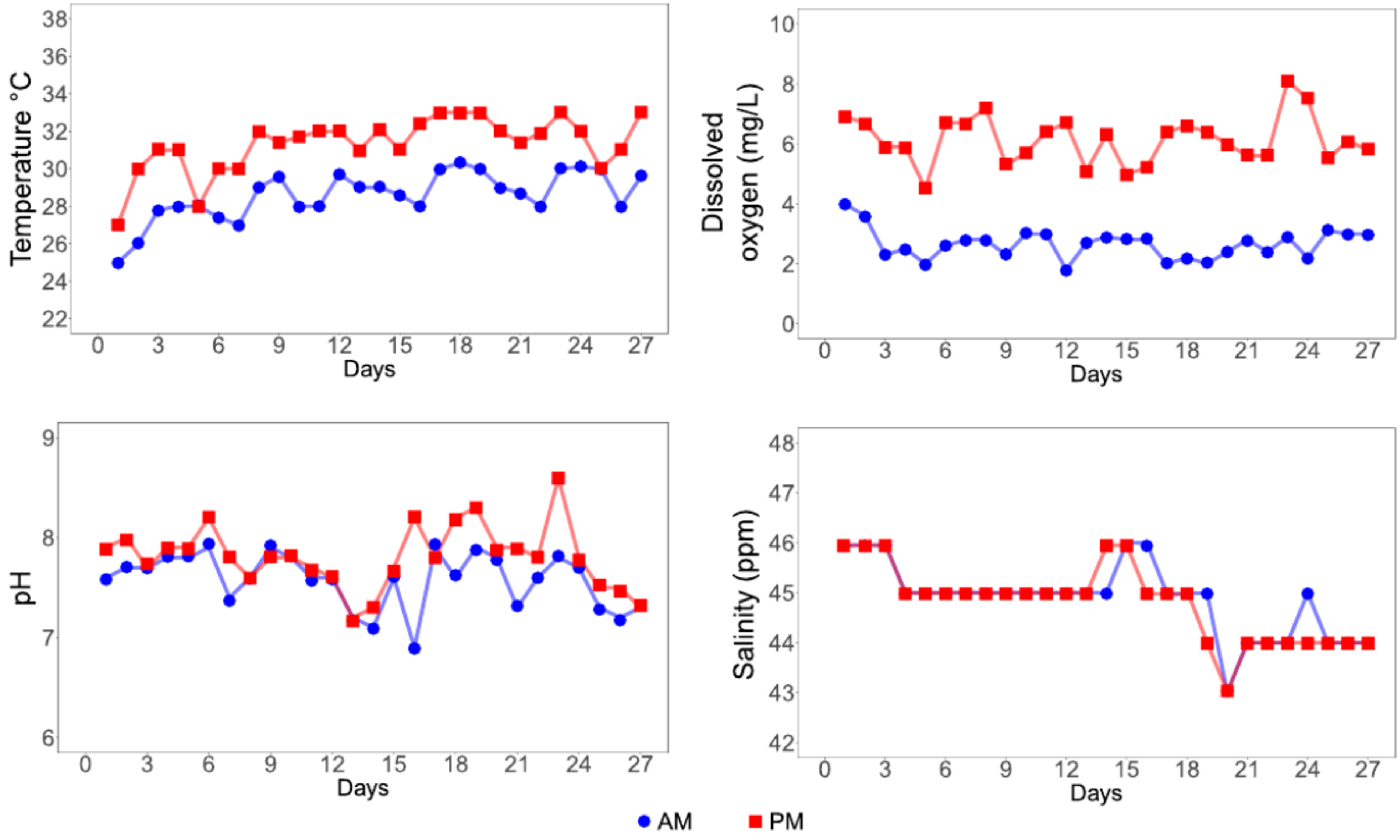

Fig. S4. Physicochemical parameters of pond water during the days of the assay. Parameters were measured twice a day, by the morning at 5 HRS (AM) and at 18 HRS (PM) in the afternoon.
