# Supplementary material for: Agavin induces beneficial microbes in the shrimp microbiota under farming conditions
Source: Sci Rep. 2022 Apr 16;12:6392. doi: 10.1038/s41598-022-10442-2 (PMC9013378; doi:10.1038/s41598-022-10442-2)
Supplement: Supplementary file 1 — Supplementary Information 1. [file 41598_2022_10442_MOESM1_ESM.zip › fig_new_S8.pdf]

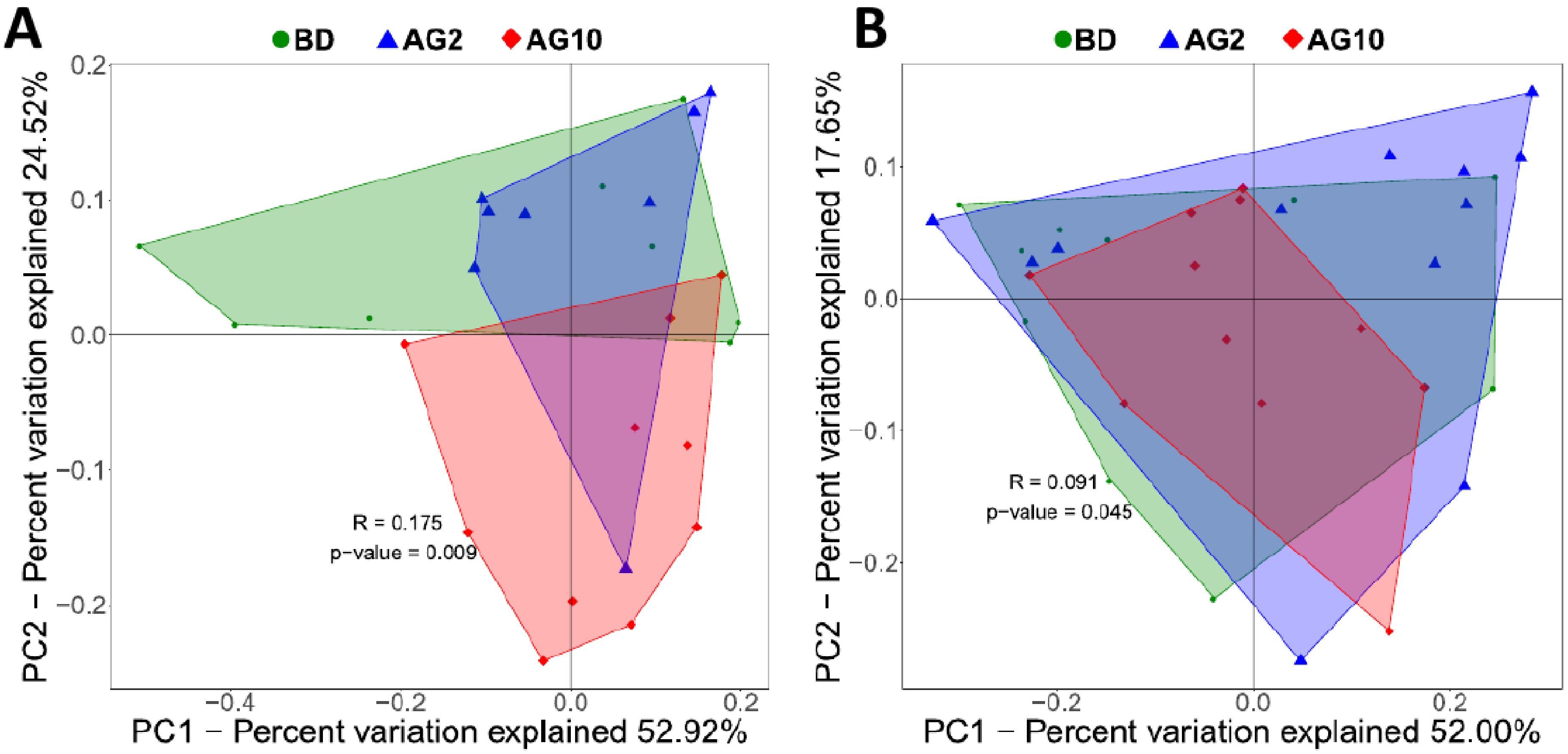

Fig. S8. The beta-diversity analysis of microbiota samples separated by each organ, hepatopancreas (A) and intestine (B), the samples in both plots was tagged by experimental diet.
