# Supplementary material for: Agavin induces beneficial microbes in the shrimp microbiota under farming conditions
Source: Sci Rep. 2022 Apr 16;12:6392. doi: 10.1038/s41598-022-10442-2 (PMC9013378; doi:10.1038/s41598-022-10442-2)
Supplement: Supplementary file 1 — Supplementary Information 1. [file 41598_2022_10442_MOESM1_ESM.zip › fig_new_s1.pdf]

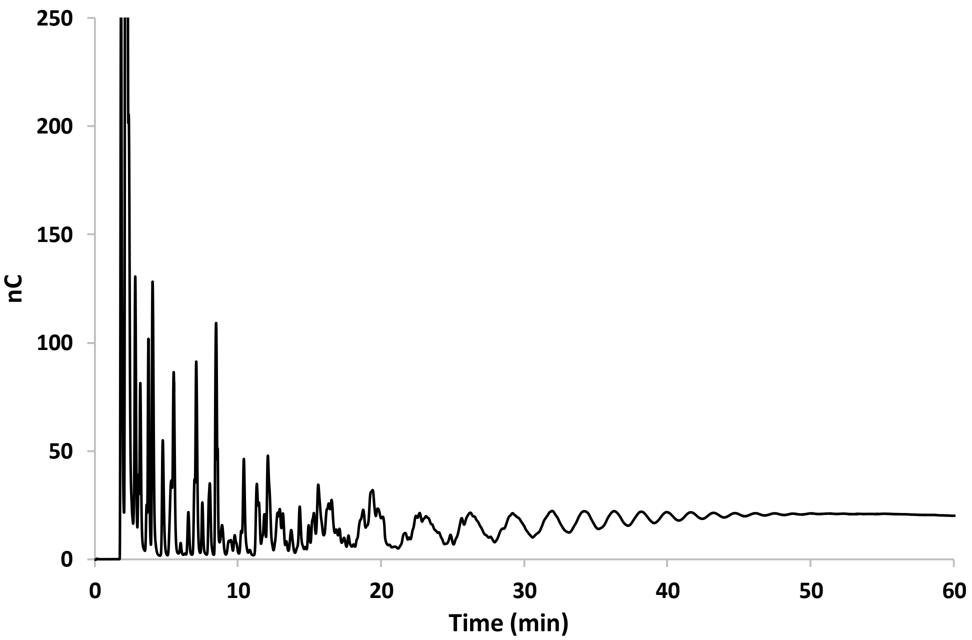

Fig. S1. Agavins extracted from agave and employed in the shrimps' diet, as observed by HPLC-SEC chromatography. As described in the text, this elution profile corresponds to fructans with 5890 Da average molecular weight, equivalent to an estimated polymerization degree of 17. See materials and methods for experimental details.
