# Supplementary material for: Agavin induces beneficial microbes in the shrimp microbiota under farming conditions
Source: Sci Rep. 2022 Apr 16;12:6392. doi: 10.1038/s41598-022-10442-2 (PMC9013378; doi:10.1038/s41598-022-10442-2)
Supplement: Supplementary file 1 — Supplementary Information 1. [file 41598_2022_10442_MOESM1_ESM.zip › fig_new_s2.pdf]

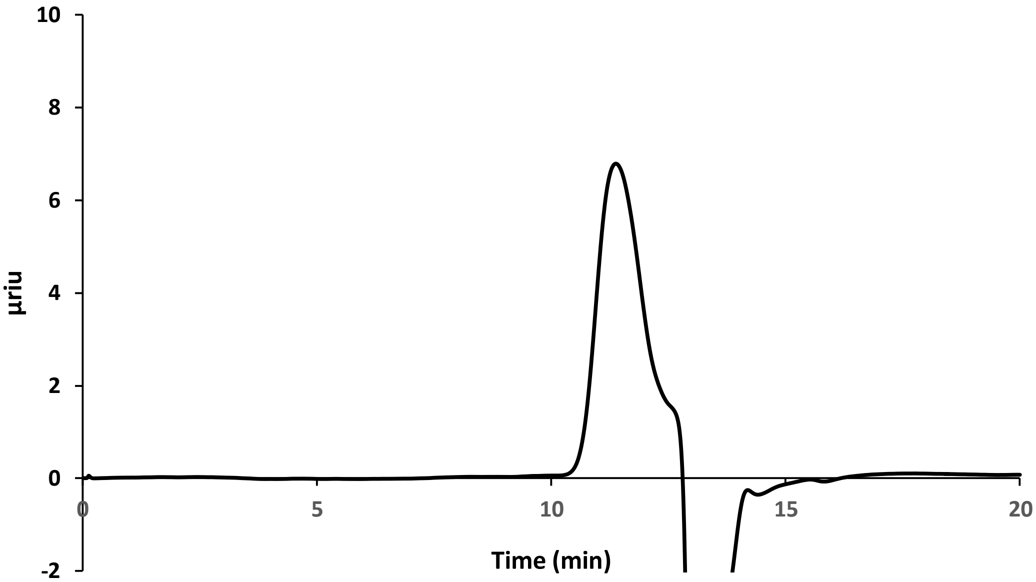

FIgure S2. Agavin oligosaccharides in the extracted agave sample, as observed by HPAEC-PAD where a wide diversity of fructooligosaccharides may be observed. This product profile is common in agavins containing linear (β2-1) and branched (β2-6) graminans and neofructans. See Material and Methods for experimental details.
