# Supplementary material for: Agavin induces beneficial microbes in the shrimp microbiota under farming conditions
Source: Sci Rep. 2022 Apr 16;12:6392. doi: 10.1038/s41598-022-10442-2 (PMC9013378; doi:10.1038/s41598-022-10442-2)
Supplement: Supplementary file 1 — Supplementary Information 1. [file 41598_2022_10442_MOESM1_ESM.zip › fig_new_s3.pdf]

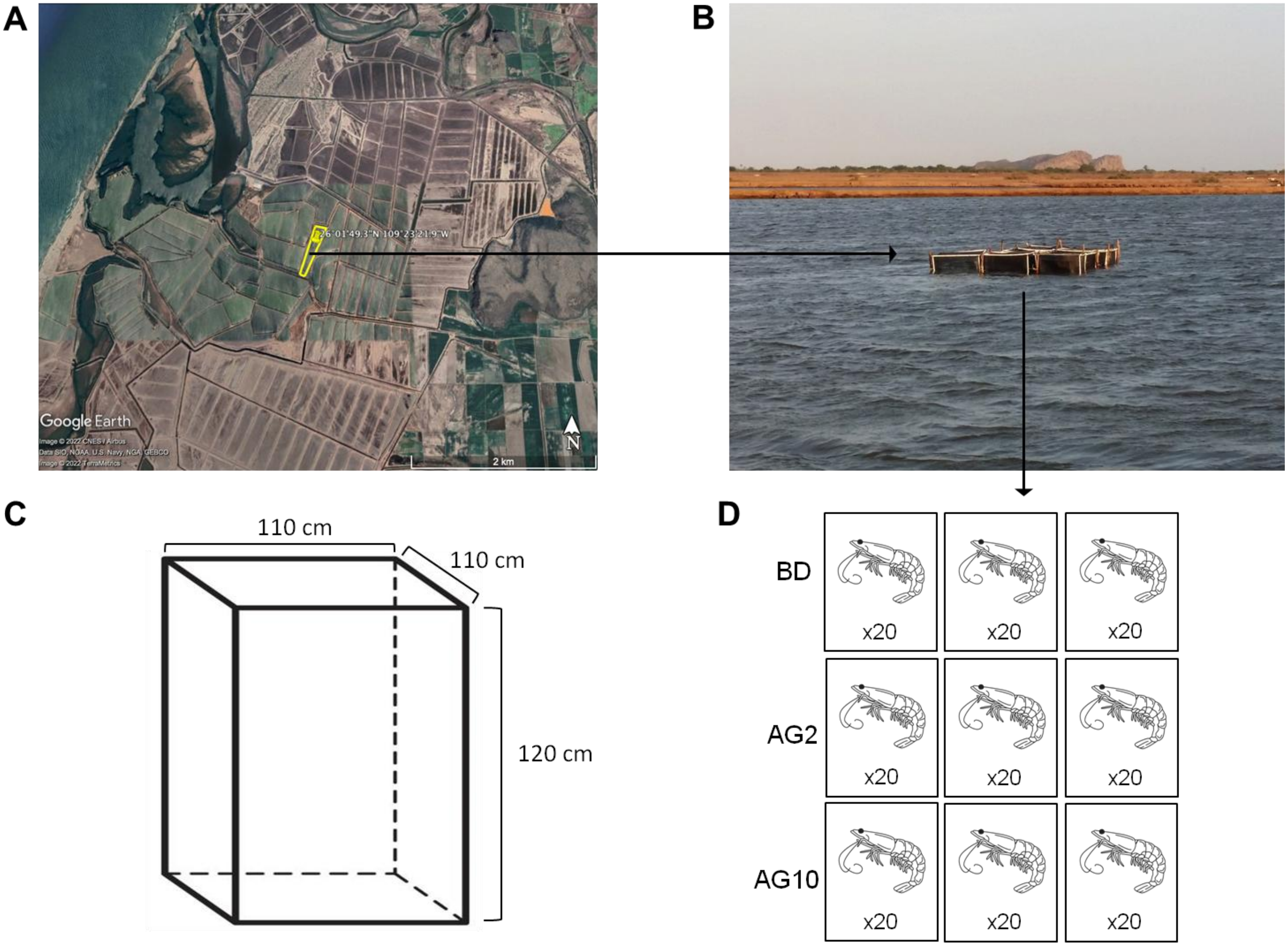

Fig. S3. Bioassay location and bioassay cage arrangement. A) Air view of shrimp farm Camarones el Renacimiento S.P.R de R.I in Northwestern Mexico. The land mark indicates the pond used for the bioassay (26º01'55.8'' N 109º23'12.4" W); B) the photograph shows all cages installed within the farm pond; C) diagram indicating the dimensions of the cages and D) diagram showing the nine cage arrangement in the pond. Three cages were assigned to each treatment (BD, AG2, and AG10)
