# Supplementary material for: Agavin induces beneficial microbes in the shrimp microbiota under farming conditions
Source: Sci Rep. 2022 Apr 16;12:6392. doi: 10.1038/s41598-022-10442-2 (PMC9013378; doi:10.1038/s41598-022-10442-2)
Supplement: Supplementary file 1 — Supplementary Information 1. [file 41598_2022_10442_MOESM1_ESM.zip › fig_new_s9.pdf]

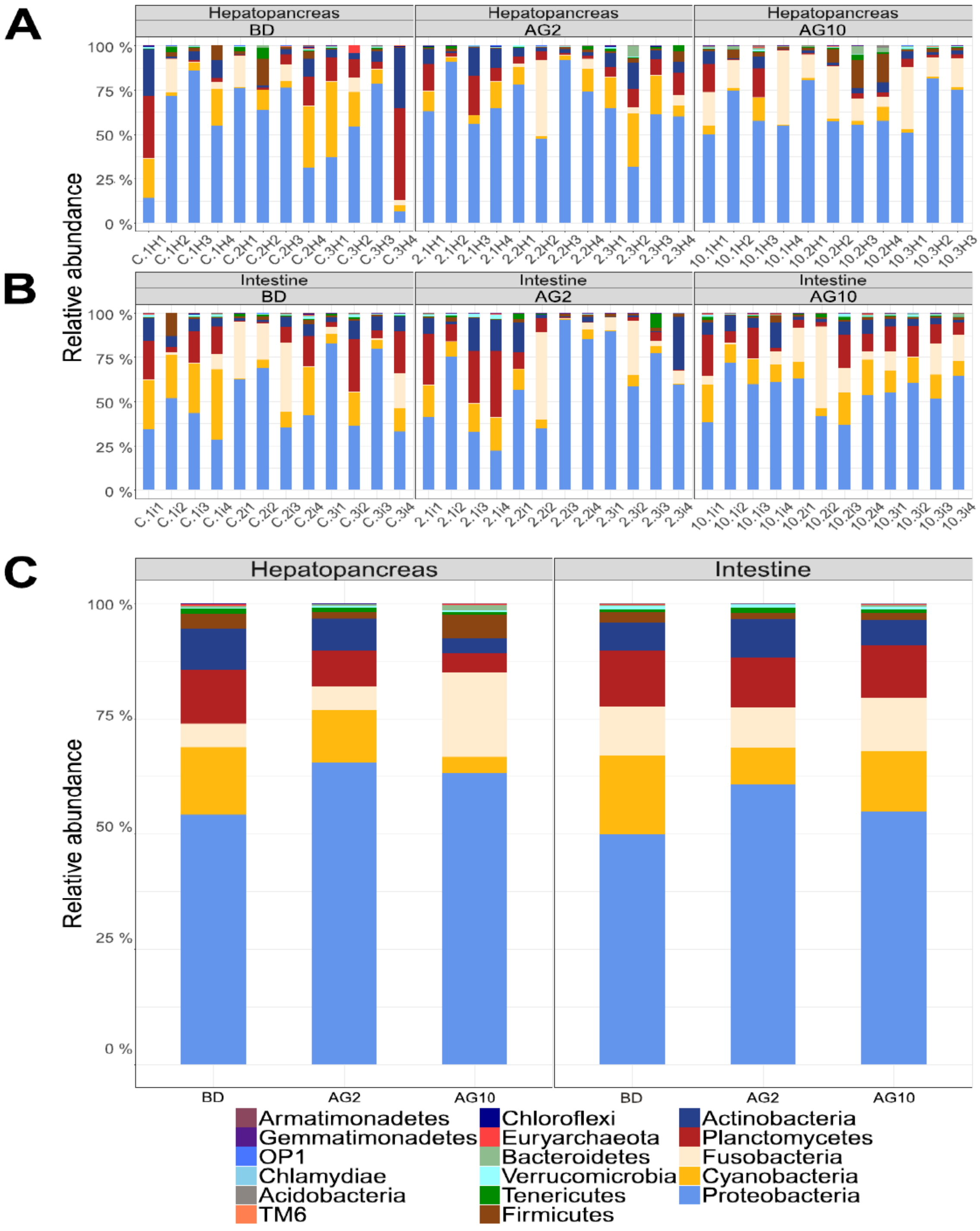

Fig. S9. Taxonomic diversity and abundance of all 35 sequenced samples at the phylum level. The Stacked-bar plot represents the relative abundance for A) hepatopancreas and B) intestine samples. C) Relative abundance in the hepatopancreas and intestine for treatment. Only the top 20 is shown; the sum of the remaining taxonomic groups is shown as "Others."
