# Supplementary figures and images for: Agavin induces beneficial microbes in the shrimp microbiota under farming conditions
Source: Sci Rep. 2022 Apr 16;12:6392. doi: 10.1038/s41598-022-10442-2 (PMC9013378; doi:10.1038/s41598-022-10442-2)

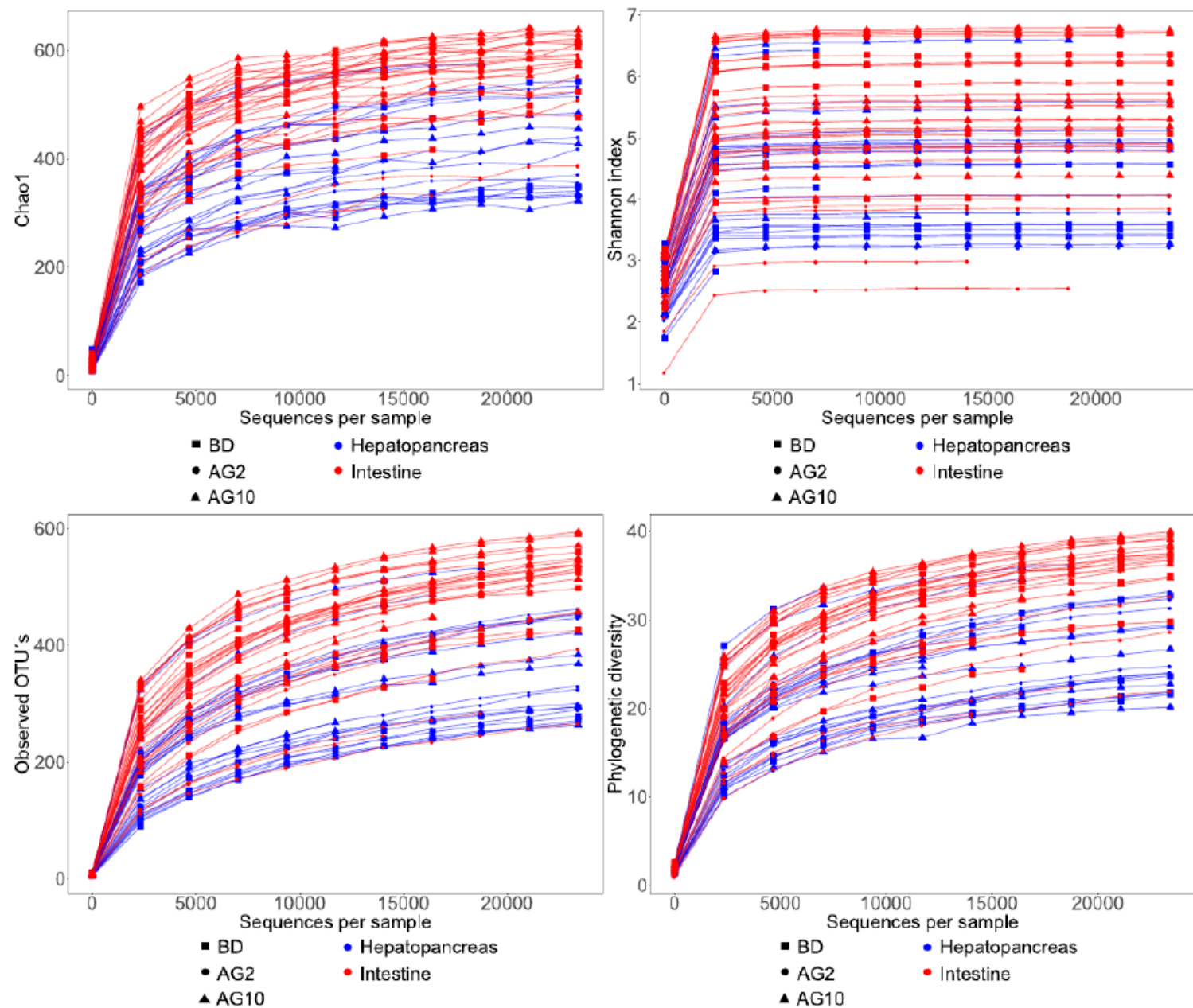

Fig. S5. Alpha diversity rarefaction curves for all samples.

Supplement: Supplementary file 1 — Supplementary Information 1. [file 41598_2022_10442_MOESM1_ESM.zip › fig_new_S5.pdf]

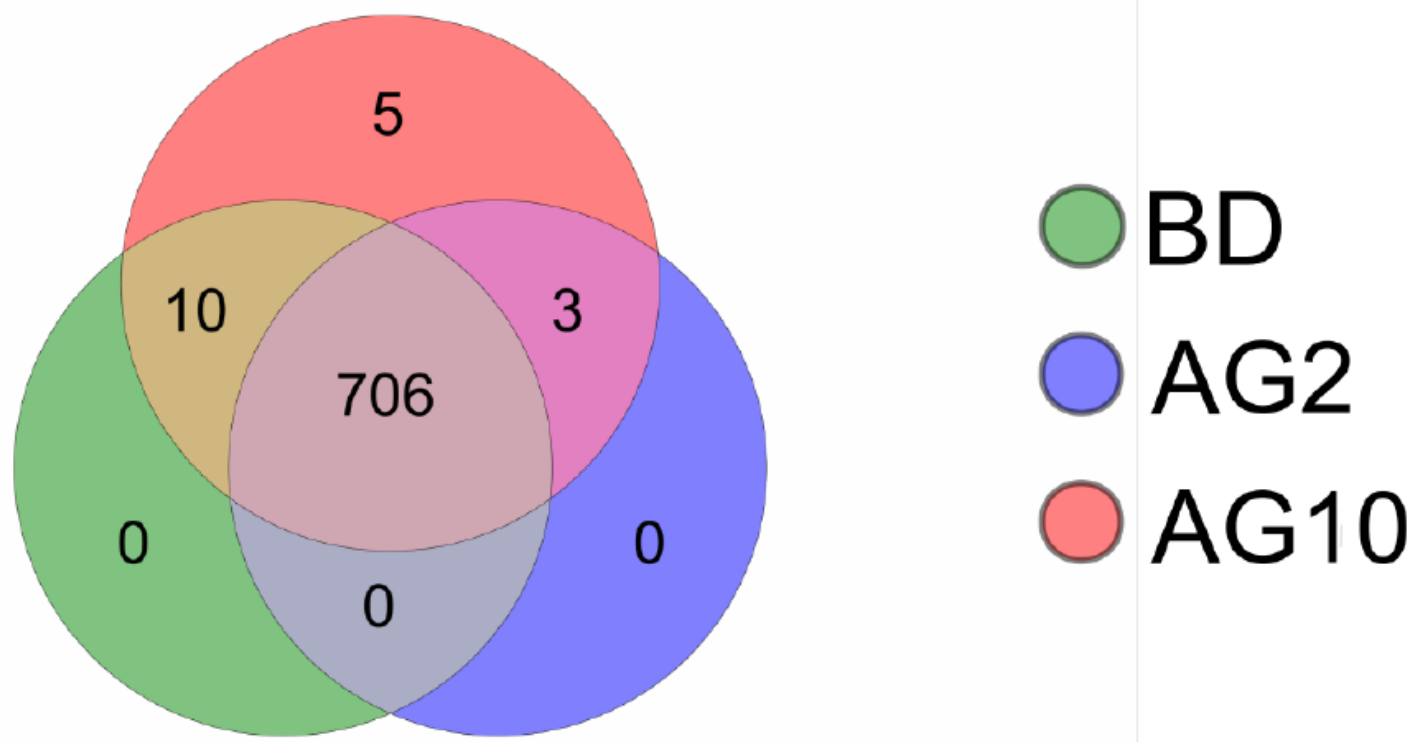

Fig. S6. Venn diagram between assigned OTUs for hepatopancreas and intestine of each treatment.

Supplement: Supplementary file 1 — Supplementary Information 1. [file 41598_2022_10442_MOESM1_ESM.zip › fig_new_S6.pdf]
