# Supplementary material for: Agavin induces beneficial microbes in the shrimp microbiota under farming conditions
Source: Sci Rep. 2022 Apr 16;12:6392. doi: 10.1038/s41598-022-10442-2 (PMC9013378; doi:10.1038/s41598-022-10442-2)
Supplement: Supplementary file 2 — Supplementary Information 2. [file 41598_2022_10442_MOESM2_ESM.zip › new_TABLE_s2.docx]

| Litopenaeus Bacteria Commensal | Penaeus Bacterial Commensal | Prawn Microbial Probiotic |
| --- | --- | --- |
| Litopenaeus Bacteria Probiont | Penaeus Bacterial Probiont | Prawn Microbial Commensal |
| Litopenaeus Bacteria Probiotic | Penaeus Bacterial Probiotic | Prawn Microbial Probiont |
| Litopenaeus Bacterial Commensal | Penaeus Microbial Probiotic | Shrimp Bacteria Commensal |
| Litopenaeus Bacterial Probiont | Penaeus Microbial Commensal | Shrimp Bacteria Probiont |
| Litopenaeus Bacterial Probiotic | Penaeus Microbial Probiont | Shrimp Bacteria Probiotic |
| Litopenaeus Microbial Probiotic | Prawn Bacteria Commensal | Shrimp Bacterial Commensal |
| Litopenaeus Microbial Commensal | Prawn Bacteria Probiont | Shrimp Bacterial Probiont |
| Litopenaeus Microbial Probiont | Prawn Bacteria Probiotic | Shrimp Bacterial Probiotic |
| Penaeus Bacteria Commensal | Prawn Bacterial Commensal | Shrimp Microbial Probiotic |
| Penaeus Bacteria Probiont | Prawn Bacterial Probiont | Shrimp Microbial Commensal |
| Penaeus Bacteria Probiotic | Prawn Bacterial Probiotic | Shrimp Microbial Probiont |

Table S2. List of keywords used for the systematic search of papers in SCOPUS.
