# Supplementary material for: Agavin induces beneficial microbes in the shrimp microbiota under farming conditions
Source: Sci Rep. 2022 Apr 16;12:6392. doi: 10.1038/s41598-022-10442-2 (PMC9013378; doi:10.1038/s41598-022-10442-2)
Supplement: Supplementary file 2 — Supplementary Information 2. [file 41598_2022_10442_MOESM2_ESM.zip › new_TABLE_s3.docx]

| **Specie identified** | **Presence of a 16S in Silva 132 database** | **Effect on shrimp health** | **Reference** |
| --- | --- | --- | --- |
| *Arthrobacter nicotianae* | Y | Antagonist properties against pathogenic *Vibrio* species | *Jayaprakash et al, 2005* |
| *Aeromonas bivalvium* | N | Improves growth, immunity response, and resistance against *Vibrio* infection | *Hao et al, 2014* |
| *Afifella marina* | Y | Improves the digestive enzyme activity, growth, and resistance against *AHPND* infection | *Chumpol et al, 2017 & 2017b* |
| *Bacillus amyloliquefaciens* | Y | Improves resistance against *Vibrio* infection | *Liu et al, 2014* |
| *Bacillus aquamaris* | N | Improves growth, immunity response, and pigmentation | *Ngo et al, 2016* |
| *Bacillus aryabhattai* | Y | Improves, immunity response, and resistance against *Vibrio* infection | *Tepaamorndech et al, 2019* |
| *Bacillus cereus* | Y | Improves growth, immunity response, and resistance against *Vibrio* infection | *Hao et al, 2014; Guo. et al, 2009* |
| *Bacillus circulans* | Y | Improves growth, survival, and overall water quality | *Nimrat et al, 2012* |
| *Bacillus coagulans* | Y | Improves the digestive enzyme activity, growth, survival, and immune response | *Zhou et al, 2009; Wang 2007; Wang & Gu, 2010* |
| *Bacillus endophyticus* | Y | Improves resistance against *Vibrio* infection | *Luis-Villaseñor et al, 2015* |
| *Bacillus firmus* | Y | Improves survival | *Pane et al, 1996; Aly et al, 2008* |
| *Bacillus flexus* | Y | Improves growth, immunity, disease resistance, and overall water quality | *Cai et al, 2019* |
| *Bacillus foraminis* | Y | Antagonist properties against pathogenic *Streptococcus* and *Photobacterium* species | *Guo. et al, 2009* |
| *Bacillus fusiformis* | Y | Improves survival | *Guo. et al, 2009* |
| *Bacillus jeotgali* | Y | Improves digestive enzyme activity | *Xue et al, 2016b* |
| *Bacillus licheniformis* | Y | Improves growth, digestive and immune enzyme activity, and overall water quality | *Li et al, 2007; Cai et al, 2019; Yuli et al, 2018* |
| *Bacillus megaterium* | Y | Improves growth, and overall water quality  Increases the abundance of beneficial bacteria in the digestive tract | *Aftabuddin et al, 2013; Nimrat et al, 2012* |
| *Bacillus polymyxa* | Y | Improves growth, survival, and overall water quality | *Nimrat et al, 2012; Yuli et al, 2018* |
| *Bacillus pumilus* | Y | Antagonist properties against pathogenic *Vibrio* species  Improves, growth, survival, pigmentation, and overall water quality  Increases the abundance of beneficial bacteria in the digestive tract | *Hill et al, 2009; Khaneja et al, 2010; Nimrat et al, 2012* |
| *Bacillus sphaericus* | Y | Antagonist properties against pathogenic *Vibrio* species  Improves growth | *Purivirojkul et al, 2006; Nguyen et al, 2014* |
| *Bacillus subtilis* | Y | Improves activity of digestive enzymes, growth, and overall water quality | *Nimrat et al, 2012; Yuli et al, 2018; Boonanuntanasarn et al, 2016* |
| *Bacillus tequilensis* | Y | Improves resistance against pathogenic *Vibrio* infection | *Luis-Villaseñor et al, 2015* |
| *Bacillus thuringiensis* | Y | Antagonist properties against pathogenic *Vibrio*  Improves the growth, and enzyme immune activity | *Ferreira et al, 2015* |
| *Bacillus vallismortis* | Y | Improves survivial, and growth  Antagonist properties against pathogenic *Vibrio* species | *Purivirojkul, 2013; Mirbakhsh et al, 2013* |
| *Bacillus vireti* | Y | Improves resistance against pathogenic *Pseudomonas* and *Aeromonas* infection | *Hindu et al, 2017 & 2018* |
| *Bacteriovorax strain DA5* | Y | Improves protection against *Vibrio* pathogen species | *Wen et al, 2014* |
| *Bdellovibrio bacteriovorus* | Y | Improves protection against pathogenic *Vibrio* and *Proteus* species | *Cao et al, 2014 & 2015* |
| *Bifidobacterium bifidum* | Y | Improves growth | *Javadi et al, 2017* |
| *Bifidobacterium thermophilum* | Y | Improves resistance against *Vibrio* pathogenic species | *Itami et al, 1998* |
| *Bowmanella denitrificans* | N | Improves resistance against pathogenic *Vibrio* species | *LaPorte et al, 2018* |
| *Clostridium butyricum* | Y | Improves digestive and immune enzyme activity, growth, survivial.  Increases epithelium height | *Duan et al, 2017, 2017b, 2018 & 2018c; Li et al, 2019; Sumon et al, 2018* |
| *Ectothiorhodospira shaposhnikovii* | N | Improves survivial, overall water quality | *Wen et al, 2015* |
| *Enterobacter hormaechei* | Y | Antagonist properties against pathogenic bacteria | *Ghosh et al, 2011* |
| *Enterococcus faecium* | Y | Improves resistance against pathogenic *Vibrio* species, survivial, and growth | *Swain et al, 2009; Sha et al, 2016; Javadi et al, 2017* |
| *Enterococcus lactis* | Y | Antagonist properties against pathogenic *Listeria* and *Pseudomonas* species | *Braïek et al, 2017, 2018, 2018b & 2018c* |
| *Exiguobacterium arabatum* | N | Production of proteins involved in the metabolism and uptake of nutrients | *Cong et al, 2017* |
| *Lactobacillus acidophilus* | Y | Improves resistence against pathogenic *Vibrio* species, growth | *Sivakumar et al, 2012; Wang & Gu, 2010; Javadi et al, 2017* |
| *Lactobacillus brevis* | Y | Antagonist properties against pathogenic *Vibrio* species | *Villamil et al, 2003; Vieira et al, 2013* |
| *Lactobacillus casei* | Y | Improves resistance against *AHPND*  Antagonist activity against pathogenic *Vibrio* species | *Villamil et al, 2003; Pinoargote et al, 2018* |
| *Lactobacillus curvatus* | N | Improves growth, and survivial | *Yuli et al, 2018* |
| *Lactobacillus delbrueckii* | Y | Antagonist properties against pathogenic bacteria  Improves growth | *Vieira et al, 2013; Javadi et al, 2017* |
| *Lactobacillus fermentum* | Y | Improves growth, resistance against pathogenic *Vibrio* species  Increases immune enzyme activity | *Wang et al, 2019* |
| *Lactobacillus graminis* | Y | Imprves growth, survivial, and immune response  Antagonist properties against pathogenic *Vibrio* species | *Bernal et al, 2017* |
| *Lactobacillus johnsonii* | Y | Improves growth,  Antagonist properties against pathogenic *Clostridium* species | *Javadi et al, 2017* |
| *Lactobacillus pentosus* | Y | Improves growth, survival, digestive enzyme activity, and resistance against infection caused by pathogenic *Vibrio* species | *Wang et al, 2019; Sha et al, 2016, 2016b & 2016c; Zheng et al, 2017; Du et al,., 2019; Javadi et al, 2017* |
| *Lactobacillus plantarum* | Y | Improves digestive and immune enzyme activity, resistance against pathogenic *Vibrio* species | *Chiu et al, 2007; Bolívar Ramírez et al, 2013; Nguyen et al, 2014; Javadi et al, 2017; Dash et al, 2014* |
| *Lactobacillus reuteri* | Y | Improves growth | *Gupta & Dhawan, 2013* |
| *Lactobacillus rhamnosus* | Y | Improves growth, immune enzyme activity. | *Sundaram et al, 2017* |
| *Lactococcus lactis* | Y | Improves growth, immune and digestive enzyme activity  Antagonist properties against pathogenic *Vibrio* species | *Adel et al, 2017; Vieira et al, 2013; Maeda et al, 2014; Loh & Ting, 2015 & 2016; Chomwong et al, 2018* |
| *Marinomonas communis* | Y | Improves survivial, water quality | *Zhang et al, 2017* |
| *Pediococcus acidilactici* | Y | Improves survivial, growth, digestive and immune enzyme activity, height of intestinal villi, resistance to infection caused by pathogenic *Vibrio* specie | *Castex et al, 2008, 2009 & 2010; Ahmadi, 2014; Boonanuntanasarn et al, 2016* |
| *Pediococcus parvulus* | N | Improves growth | *Apún-Molina et al, 2015* |
| *Pediococcus pentosaceus* | Y | Improves growth, survival, digestive and immune enzyme activity, resistance against *AHPND* and *WSSV* | *Adel et al, 2017b; Leyva-Madrigal et al, 2011* |
| *Phaeobacter gallaeciensis* | N | Improves growth  Antagonist properties against pathogenic *Vibrio* species | *Balcázar et al, 2007* |
| *Phaeobacter inhibens* | N | Antagonist properties against pathogenic *Vibrio* species | *Grotkjær et al, 2016* |
| *Pseudoalteromonas flavipulchra* | Y | Improves survivial | *Xue et al, 2016* |
| *Pseudoalteromonas piscicida* | Y | Improves nutrient uptake, growth, survivial  Antagonist properties against pathogenic bacteria | *Yuhana et al, 2017; Sánchez-Díaz et al, 2019; Louis et al, 2018* |
| *Pseudoalteromonas ruthenica* | Y | Production of digestive enzymes | *Li et al, 2011* |
| *Pseudomonas aeruginosa* | Y | Antagonist properties against pathogenic *Vibrio* species  Improves growth | *Van et al, 2007 & 2009; Hai et al, 2009, 2010 & 2010b; Chau et al, 2011; Chythanya et al, 2002; Vijayan et al, 2006* |
| *Pseudomonas putida* | Y | Improves innate immunity. | *Jakhar et al, 2016* |
| *Pseudomonas synxantha* | Y | Improves growth, and innate immune response.  Antagonist properties against pathogenic *Vibrio* species | *Van et al, 2007 & 2009; Hai et al, 2009, 2010 & 2010b* |
| *Rhodobacter capsulatus* | Y | Production of digestive enzymes | *Dou et al, 2016* |
| *Rhodobacter sphaeroides* | Y | Improves growth, overall water quality, resistance against *AHPND* infection, | *Chumpol et al, 2017 & 2017b* |
| *Rhodopseudomonas palustris* | Y | Improves growth, survivial, immune ezyme activity, resistance against *AHPND* infection | *Wang & Gu, 2010; Wen et al, 2015; Pinoargote et al, 2018* |
| *Shewanella algae* | Y | Antagonist properties against pathogenic Vibrio species | *Shakibazadeh et al, 2008 & 2011; Zadeh et al, 2010; Interaminense et al, 2018 & 2019* |
| *Shewanella haliotis* | Y | Improves, growth, survival, immune enzyme activity, resistance against pathogenic *Vibrio* infection | *Hao et al, 2014* |
| *Staphylococcus haemolyticus* | Y | Protects against of *WSSV* infection. | *Leyva-Madrigal et al, 2011* |
| *Streptococcus phocae* | Y | Improves survival  Antagonist properties against pathogenic *Vibrio*, and *Listeria* species. | *Swain et al, 2009; Satish & Arul, 2009; Pattukumar et al, 2013 & 2014; Kanmmani et al, 2010* |
| *Streptococcus salivarius* | Y | Improves growth, and survival | *Najmi et al, 2018* |
| *Streptococcus thermophilus* | Y | Improves growth | *Javadi et al, 2017* |
| *Streptomyces californicus* | N | Antagonist properties against pathogenic *Vibrio* species | *Gozari et al, 2016* |
| *Streptomyces fradiae* | Y | Improves growth, and nutrient uptake | *Aftabuddin et al, 2013* |
| *Streptomyces griseus* | Y | Antagonist properties against pathogenic *Vibrio* species | *You et al, 2005* |
| *Streptomyces rubrolavendulae* | Y | Antagonist properties against pathogenic *Vibrio* species | *Augustine et al, 2016* |
| Vibrio alginolyticus | Y | Antagonist activity against pathogenic Vibrio species. | *Gullian et al, 2004; Thompson et al, 2010; Austin et al, 1995; Gomez-Gil et al, 2002* |
| Vibrio fluvialis | Y | Improves survivial | *Alavandi et al, 2004; El-Sersy et al, 2006* |
| *Vibrio gazogenes* | Y | Antagonist activity against pathogenic bacteria | *Thompson et al, 2010* |
| *Vibrio hepatarius* | Y | Improves resistance against pathogenic *Vibrio* infection | *Gullian et al, 2004* |
| *Vibrio mediterranei* | Y | Antagonist properties against pathogenic *Vibrio* species | *Carraturo et al, 2006* |
| *Weissella confusa* | Y | Antagonist properties against pathogenic bacteria | *Vieira et al, 2013* |

Table S3. List of probiotic species identified in the literature. The “Y” indicates a 16S sequence for that specie in Silva132. The “N” indicates that the species is not present in the Silva132 database.

References

- Adel, M., El-Sayed, A. F. M., Yeganeh, S., Dadar, M., & Giri, S. S. (2017). Effect of potential probiotic Lactococcus lactis subsp. lactis on growth performance, intestinal microbiota, digestive enzyme activities, and disease resistance of Litopenaeus vannamei. Probiotics and antimicrobial proteins, 9(2), 150-156.
- Adel, M., Yeganeh, S., Dawood, M. A. O., Safari, R., & Radhakrishnan, S. (2017b). Effects of Pediococcus pentosaceus supplementation on growth performance, intestinal microflora and disease resistance of white shrimp, Litopenaeus vannamei. Aquaculture Nutrition, 23(6), 1401-1409.
- Aftabuddin, S., Kashem, M. A., Kader, M. A., Sikder, M., & Hakim, M. A. (2013). Use of Streptomyces fradiae and Bacillus megaterium as probiotics in the experimental culture of tiger shrimp Penaeus monodon (Crustacea, Penaeidae). Aquaculture, Aquarium, Conservation & Legislation-International Journal of the Bioflux Society (AACL Bioflux), 6(3).
- Ahmadi, S. (2014). Comparative effect of Pediococcus acidilactici and Lactococcus lactis on growth performance, survival and enzyme activity of western white leg shrimp (Litopenaeus vannamei) (Doctoral dissertation, Islamic Azad University, Science and Research Branch, Tehran).
- Alavandi, S. V., Vijayan, K. K., Santiago, T. C., Poornima, M., Jithendran, K. P., Ali, S. A., & Rajan, J. J. S. (2004). Evaluation of Pseudomonas sp. PM 11 and Vibrio fluvialis PM 17 on immune indices of tiger shrimp, Penaeus monodon. Fish & Shellfish Immunology, 17(2), 115-120.
- Aly, S. M., Abd-El-Rahman, A. M., John, G., & Mohamed, M. F. (2008). Characterization of some bacteria isolated from Oreochromis niloticus and their potential use as probiotics. Aquaculture, 277(1-2), 1-6.
- Apún-Molina, J. P., Santamaría-Miranda, A., Luna-González, A., Ibarra-Gámez, J. C., Medina-Alcantar, V., & Racotta, I. (2015). Growth and metabolic responses of whiteleg shrimp Litopenaeus vannamei and Nile tilapia Oreochromis niloticus in polyculture fed with potential probiotic microorganisms on different schedules: Crecimiento y respuesta metabólica del camarón blanco Litopenaeus vannamei y tilapia del Nilo Oreochromis niloticus en policultivo alimentado con microorganismos probióticos potenciales en diferentes frecuencias. Latin american journal of aquatic research, 43(3), 435-445.
- Augustine, D., Jacob, J. C., & Philip, R. (2016). Exclusion of Vibrio spp. by an antagonistic marine actinomycete Streptomyces rubrolavendulae M56. Aquaculture research, 47(9), 2951-2960.
- Austin, B., Stuckey, L. F., Robertson, P. A. W., Effendi, I., & Griffith, D. R. W. (1995). A probiotic strain of Vibrio alginolyticus effective in reducing diseases caused by Aeromonas salmonicida, Vibrio anguillarum and Vibrio ordalii. Journal of Fish Diseases, 18(1), 93-96.
- Balcázar, J. L., Rojas-Luna, T., & Cunningham, D. P. (2007). Effect of the addition of four potential probiotic strains on the survival of pacific white shrimp (Litopenaeus vannamei) following immersion challenge with Vibrio parahaemolyticus. Journal of invertebrate pathology, 96(2), 147-150.
- Bernal, M. G., Marrero, R. M., Campa-Córdova, Á. I., & Mazón-Suástegui, J. M. (2017). Probiotic effect of Streptomyces strains alone or in combination with Bacillus and Lactobacillus in juveniles of the white shrimp Litopenaeus vannamei. Aquaculture International, 25(2), 927-939.
- Bolívar Ramírez, N., Seiffert, W. Q., Vieira, F. D. N., Mouriño, J. L. P., Jesus, G. F. A., Ferreira, G. S., & Andreatta, E. R. (2013). Prebiotic, probiotic, and symbiotic-supplemented diet for marine shrimp farming. Pesquisa Agropecuária Brasileira, 48(8), 913-919.
- Boonanuntanasarn, S., Wongsasak, U., Pitaksong, T., & Chaijamrus, S. (2016). Effects of dietary supplementation with b-glucan and synbiotics on growth, haemolymph chemistry, and intestinal microbiota and morphology in the P acific white shrimp. Aquaculture nutrition, 22(4), 837-845.
- Braïek, O. B., Cremonesi, P., Morandi, S., Smaoui, S., Hani, K., & Ghrairi, T. (2018c). Safety characterisation and inhibition of fungi and bacteria by a novel multiple enterocin-producing Enterococcus lactis 4CP3 strain. Microbial pathogenesis, 118, 32-38.
- Braïek, O. B., Ghomrassi, H., Cremonesi, P., Morandi, S., Fleury, Y., Le Chevalier, P., ... & Ghrairi, T. (2017). Isolation and characterisation of an enterocin P-producing Enterococcus lactis strain from a fresh shrimp (Penaeus vannamei). Antonie van Leeuwenhoek, 110(6), 771-786.
- Braïek, O. B., Morandi, S., Cremonesi, P., Smaoui, S., Hani, K., & Ghrairi, T. (2018). Biotechnological potential, probiotic and safety properties of newly isolated enterocin-producing Enterococcus lactis strains. LWT, 92, 361-370.
- Braïek, O. B., Morandi, S., Cremonesi, P., Smaoui, S., Hani, K., & Ghrairi, T. (2018b). Safety, potential biotechnological and probiotic properties of bacteriocinogenic Enterococcus lactis strains isolated from raw shrimps. Microbial pathogenesis, 117, 109-117.
- Cai, Y., Yuan, W., Wang, S., Guo, W., Li, A., Wu, Y., ... & Zhou, Y. (2019). In vitro screening of putative probiotics and their dual beneficial effects: To white shrimp (Litopenaeus vannamei) postlarvae and to the rearing water. Aquaculture, 498, 61-71.
- Cao, H., An, J., Zheng, W., & He, S. (2015). Vibrio cholerae pathogen from the freshwater-cultured whiteleg shrimp Penaeus vannamei and control with Bdellovibrio bacteriovorus. Journal of invertebrate pathology, 130, 13-20.
- Cao, H., He, S., Lu, L., Yang, X., & Chen, B. (2014). Identification of a Proteus penneri isolate as the causal agent of red body disease of the cultured white shrimp Penaeus vannamei and its control with Bdellovibrio bacteriovorus. Antonie van Leeuwenhoek, 105(2), 423-430.
- Carraturo, A., Raieta, K., Ottaviani, D., & Russo, G. L. (2006). Inhibition of Vibrio parahaemolyticus by a bacteriocin like inhibitory substance (BLIS) produced by Vibrio mediterranei 1. Journal of applied microbiology, 101(1), 234-241.
- Castex, M., Chim, L., Pham, D., Lemaire, P., Wabete, N., Nicolas, J. L., ... & Mariojouls, C. (2008). Probiotic P. acidilactici application in shrimp Litopenaeus stylirostris culture subject to vibriosis in New Caledonia. Aquaculture, 275(1-4), 182- 193.
- Castex, M., Lemaire, P., Wabete, N., & Chim, L. (2009). Effect of dietary probiotic Pediococcus acidilactici on antioxidant defences and oxidative stress status of shrimp Litopenaeus stylirostris. Aquaculture, 294(3-4), 306-313.
- Castex, M., Lemaire, P., Wabete, N., & Chim, L. (2010). Effect of probiotic Pediococcus acidilactici on antioxidant defences and oxidative stress of Litopenaeus stylirostris under Vibrio nigripulchritudo challenge. Fish & shellfish immunology, 28(4), 622-631.
- Chau, N. T. T., Quang, P. H., Lan, P. T. N., Matsumoto, M., & Miyajima, I. (2011). Identification and Characterization of Pseudomonas sp. P 9 Antagonistic to Pathogenic Vibrio spp. Isolated from Shrimp Culture Pond in Thua Thien Hue-VietNam. Journal of the Faculty of Agriculture, Kyushu University, 56(1), 23-31.
- Chiu, C. H., Guu, Y. K., Liu, C. H., Pan, T. M., & Cheng, W. (2007). Immune responses and gene expression in white shrimp, Litopenaeus vannamei, induced by Lactobacillus plantarum. Fish & Shellfish Immunology, 23(2), 364-377.
- Chomwong, S., Charoensapsri, W., Amparyup, P., & Tassanakajon, A. (2018). Two host gut-derived lactic acid bacteria activate the proPO system and increase resistance to an AHPND-causing strain of Vibrio parahaemolyticus in the shrimp Litopenaeus vannamei. Developmental & Comparative Immunology, 89, 54-65.
- Chumpol, S., Kantachote, D., Nitoda, T., & Kanzaki, H. (2017). The roles of probiotic purple nonsulfur bacteria to control water quality and prevent acute hepatopancreatic necrosis disease (AHPND) for enhancement growth with higher survival in white shrimp (Litopenaeus vannamei) during cultivation. Aquaculture, 473, 327-336.
- Chumpol, S., Kantachote, D., Rattanachuay, P., Vuddhakul, V., Nitoda, T., & Kanzaki, H. (2017). In vitro and in vivo selection of probiotic purple nonsulphur bacteria with an ability to inhibit shrimp pathogens: acute hepatopancreatic necrosis disease‐causing Vibrio parahaemolyticus and other vibrios. Aquaculture research, 48(6), 3182-3197.
- Chythanya, R., Karunasagar, I., & Karunasagar, I. (2002). Inhibition of shrimp pathogenic vibrios by a marine Pseudomonas I-2 strain. Aquaculture, 208(1-2), 1-10.
- Cong, M., Jiang, Q., Xu, X., Huang, L., Su, Y., & Yan, Q. (2017). The complete genome sequence of Exiguobacterium arabatum W-01 reveals potential probiotic functions. MicrobiologyOpen, 6(5), e00496.
- Dash, G., Raman, R. P., Prasad, K. P., Makesh, M., Pradeep, M. A., & Sen, S. (2014). Evaluation of Lactobacillus plantarum as feed supplement on host associated microflora, growth, feed efficiency, carcass biochemical composition and immune response of giant freshwater prawn, Macrobrachium rosenbergii (de Man, 1879). Aquaculture, 432, 225-236.
- Dou, C., Zuo, Z., Liu, Y., Zhang, Y., Geng X, X., & Sun, J. (2016). Isolation and screening of digestive enzyme producing probiotics from intestine of Litopenaeus vannamei. Journal of Fisheries of China, 40, 537.
- Du, Y., Zhou, S., Liu, M., Wang, B., Jiang, K., Fang, H., & Wang, L. (2019). Understanding the roles of surface proteins in regulation of Lactobacillus pentosus HC-2 to immune response and bacterial diversity in midgut of Litopenaeus vannamei. Fish & shellfish immunology, 86, 1194-1206.
- Duan, Y., Dong, H., Wang, Y., Zhang, Y., & Zhang, J. (2018). Effects of the dietary probiotic Clostridium butyricum on intestine digestive and metabolic capacities, SCFA content and body composition in Marsupenaeus japonicus. Journal of Ocean University of China, 17(3), 690-696.
- Duan, Y., Wang, Y., Dong, H., & Zhang, J. S. (2018b). Changes in the intestine microbial, digestive and immune-related genes of Litopenaeus vannamei in response to dietary probiotic Clostridium butyricum supplementation. Frontiers in microbiology, 9, 2191
- Duan, Y., Zhang, Y., Dong, H., Wang, Y., & Zhang, J. (2017). Effect of the dietary probiotic Clostridium butyricum on growth, intestine antioxidant capacity and resistance to high temperature stress in kuruma shrimp Marsupenaeus japonicus. Journal of thermal biology, 66, 93-100.
- Duan, Y., Zhang, Y., Dong, H., Wang, Y., Zheng, X., & Zhang, J. (2017b). Effect of dietary Clostridium butyricum on growth, intestine health status and resistance to ammonia stress in Pacific white shrimp Litopenaeus vannamei. Fish & shellfish immunology, 65, 25-33
- El-Sersy, N. A., AbdelRazek, F. A., & Taha, S. M. (2006). Evaluation of various probiotic bacteria for the survival of Penaeus japonicus larvae. Fresenius Environmental Bulletin, 15(12), 1506.
- Ferreira, G. S., Bolívar, N. C., Pereira, S. A., Guertler, C., do Nascimento Vieira, F., Mouriño, J. L. P., & Seiffert, W. Q. (2015). Microbial biofloc as source of probiotic bacteria for the culture of Litopenaeus vannamei. Aquaculture, 448, 273-279.
- Ghosh, S., Ringø, E., Deborah, G. S. A., Rahiman, K. M., & Hatha, A. A. M. (2011). Enterobacter hormaechei bac 1010 from the gut of flathead grey mullet as probable aquaculture probiont. Journal of Nature Science and Sustainable Technology, 5(3), 189.
- Gomez-Gil, B., Roque, A., & Velasco-Blanco, G. (2002). Culture of Vibrio alginolyticus C7b, a potential probiotic bacterium, with the microalga Chaetoceros muelleri. Aquaculture, 211(1-4), 43-48.
- Gozari, M., Mortazavi, M. S., Bahador, N., & Rabbaniha, M. (2016). Isolation and screening of antibacterial and enzyme producing marine actinobacteria to approach probiotics against some pathogenic vibrios in shrimp Litopenaeus vannamei. Iranian Journal of Fisheries Sciences, 15(2), 630-644.
- Grotkjær, T., Bentzon-Tilia, M., D'Alvise, P., Dierckens, K., Bossier, P., & Gram, L. (2016). Phaeobacter inhibens as probiotic bacteria in non-axenic Artemia and algae cultures. Aquaculture, 462, 64-69.
- Gullian, M., Thompson, F., & Rodriguez, J. (2004). Selection of probiotic bacteria and study of their immunostimulatory effect in Penaeus vannamei. Aquaculture, 233(1-4), 1-14.
- Guo, J. J., Liu, K. F., Cheng, S. H., Chang, C. I., Lay, J. J., Hsu, Y. O., ... & Chen, T. I. (2009). Selection of probiotic bacteria for use in shrimp larviculture. Aquaculture Research, 40(5), 609-618.
- Gupta, A., & Dhawan, A. (2013). Probiotic based diets for freshwater prawn Macrobrachium rosenbergii (de Man). Indian Journal of Fisheries, 60(1), 103-109.
- Hai, N. V., Buller, N., & Fotedar, R. (2009). Effects of probiotics (Pseudomonas synxantha and Pseudomonas aeruginosa) on the growth, survival and immune parameters of juvenile western king prawns (Penaeus latisulcatus Kishinouye, 1896). Aquaculture Research, 40(5), 590-602.
- Hai, N. V., Buller, N., & Fotedar, R. (2010). Effect of customized probiotics on the physiological and immunological responses of juvenile western king prawns (Penaeus latisulcatus Kishinouye, 1896) challenged with Vibrio harveyi. Journal of Applied Aquaculture, 22(4), 321-336.
- Hai, N., Buller, N., & Fotedar, R. (2010b). Encapsulation capacity of Artemia nauplii with customized probiotics for use in the cultivation of western king prawns (Penaeus latisulcatus Kishinouye, 1896).
- Hao, K., Liu, J. Y., Ling, F., Liu, X. L., Lu, L., Xia, L., & Wang, G. X. (2014). Effects of dietary administration of Shewanella haliotis D4, Bacillus cereus D7 and Aeromonas bivalvium D15, single or combined, on the growth, innate immunity and disease resistance of shrimp, Litopenaeus vannamei. Aquaculture, 428, 141-149.
- Hill, J. E., Baiano, J. C. F., & Barnes, A. C. (2009). Isolation of a novel strain of Bacillus pumilus from penaeid shrimp that is inhibitory against marine pathogens. Journal of Fish diseases, 32(12), 1007-1016.
- Hindu, S. V., Chandrasekaran, N., Mukherjee, A., & Thomas, J. (2017). Effect of dietary supplementation of novel probiotic bacteria Bacillus vireti 01 on antioxidant defence system of freshwater prawn challenged with Pseudomonas aeruginosa. Probiotics and antimicrobial proteins, 1-11.
- Hindu, S. V., Thanigaivel, S., Vijayakumar, S., Chandrasekaran, N., Mukherjee, A., & Thomas, J. (2018). Effect of microencapsulated probiotic Bacillus vireti 01-polysaccharide extract of Gracilaria folifera with alginate-chitosan on immunity, antioxidant activity and disease resistance of Macrobrachium rosenbergii against Aeromonas hydrophila infection. Fish & shellfish immunology, 73, 112-120.
- Interaminense, J. A., Vogeley, J. L., Gouveia, C. K., Portela, R. S., Oliveira, J. P., Silva, S. M., ... & Bezerra, R. S. (2019). Effects of dietary Bacillus subtilis and Shewanella algae in expression profile of immune-related genes from hemolymph of Litopenaeus vannamei challenged with Vibrio parahaemolyticus. Fish & shellfish immunology, 86, 253-259.
- Interaminense, J. A., Vogeley, J. L., Gouveia, C. K., Portela, R. W., Oliveira, J. P., Andrade, H. A., ... & Bezerra, R. S. (2018). In vitro and in vivo potential probiotic activity of Bacillus subtilis and Shewanella algae for use in Litopenaeus vannamei rearing. Aquaculture, 488, 114-122.
- Itami, T., Asano, M., Tokushige, K., Kubono, K., Nakagawa, A., Takeno, N., ... & Takahashi, Y. (1998). Enhancement of disease resistance of kuruma shrimp, Penaeus japonicus, after oral administration of peptidoglycan derived from Bifidobacterium thermophilum. Aquaculture, 164(1-4), 277-288.
- Jakhar, V., Sihag, R. C., & Gahlawat, S. K. (2016). Effect of probiotics on immunological status of giant freshwater prawn (macrobrachium rosenbergii de Man). Indian Journal of Animal Research, 50(6), 930-935.
- Javadi, A., & Khatibi, S. A. (2017). Effect of commercial probiotic (Protexin®) on growth, survival and microbial quality of shrimp (Litopenaeus vannamei). Nutrition & Food Science, 47(2), 204-216
- Jayaprakash, N. S., Pai, S. S., Anas, A., Preetha, R., Philip, R., & Singh, I. B. (2005). A marine bacterium, Micrococcus MCCB 104, antagonistic to vibrios in prawn larval rearing systems. Diseases of Aquatic Organisms, 68(1), 39-45.
- Khaneja, R., Perez-Fons, L., Fakhry, S., Baccigalupi, L., Steiger, S., To, E., ... & Cutting, S. M. (2010). Carotenoids found in Bacillus. Journal of applied microbiology, 108(6), 1889-1902.
- LaPorte, J. P., Spinard, E. J., Gomez-Chiarri, M., Rowley, D. C., Mekalanos, J. J., & Nelson, D. R. (2018). Draft Genome Sequence of Bowmanella denitrificans JL63, a Bacterium Isolated from Whiteleg Shrimp (Litopenaeus vannamei) That Can Inhibit the Growth of Vibrio parahaemolyticus. Genome announcements, 6(14), e00215-18.
- Leyva-Madrigal, K. Y., Luna-González, A., Escobedo-Bonilla, C. M., Fierro-Coronado, J. A., & Maldonado-Mendoza, I. E. (2011). Screening for potential probiotic bacteria to reduce prevalence of WSSV and IHHNV in whiteleg shrimp (Litopenaeus vannamei) under experimental conditions. Aquaculture, 322, 16-22.
- Li, H., Tian, X., Zhao, K., Jiang, W., & Dong, S. (2019). Effect of Clostridium butyricum in different forms on growth performance, disease resistance, expression of genes involved in immune responses and mTOR signaling pathway of Litopenaeus vannamai. Fish & shellfish immunology, 87, 13-21.
- Li, J., Tan, B., & Mai, K. (2011). Isolation and identification of a bacterium from marine shrimp digestive tract: A new degrader of starch and protein. Journal of Ocean University of China, 10(3), 287-292.
- Li, K., Zheng, T., Tian, Y., Xi, F., Yuan, J., Zhang, G., & Hong, H. (2007). Beneficial effects of Bacillus licheniformis on the intestinal microflora and immunity of the white shrimp, Litopenaeus vannamei. Biotechnology Letters, 29(4), 525-530.
- Liu, H., Li, Z., Tan, B., Lao, Y., Duan, Z., Sun, W., & Dong, X. (2014). Isolation of a putative probiotic strain S12 and its effect on growth performance, non-specific immunity and disease-resistance of white shrimp, Litopenaeus vannamei. Fish & shellfish immunology, 41(2), 300-307.
- Loh, J. Y., & Ting, A. S. Y. (2015). Bioencapsulation of probiotic Lactococcus lactis subsp. lactis on Artemia franciscana nauplii: effects of encapsulation media on nauplii survival and probiotic recovery. Malaysian Journal of Microbiology, 11(2), 121-127.
- Loh, J. Y., & Ting, A. S. Y. (2016). Effects of potential probiotic Lactococcus lactis subsp. lactis on digestive enzymatic activities of live feed Artemia franciscana. Aquaculture international, 24(5), 1341-1351.
- Louis, S., Nelly, W., Dominique, A., Jean-René, M., Marie, P., Cong, Z., ... & Dominique, P. (2018). Survival improvement conferred by the Pseudoalteromonas sp. NC201 probiotic in Litopenaeus stylirostris exposed to Vibrio nigripulchritudo infection and salinity stress. Aquaculture, 495, 888-898.
- Luis-Villaseñor, I. E., Voltolina, D., Gomez-Gil, B., Ascencio, F., Campa-Córdova, Á. I., Audelo-Naranjo, J. M., & Zamudio-Armenta, O. O. (2015). Probiotic modulation of the gut bacterial community of juvenile Litopenaeus vannamei challenged with Vibrio parahaemolyticus CAIM 170. Latin American Journal of Aquatic Research, 43(4).
- Maeda, M., Shibata, A., Biswas, G., Korenaga, H., Kono, T., Itami, T., & Sakai, M. (2014). Isolation of lactic acid bacteria from kuruma shrimp (Marsupenaeus japonicus) intestine and assessment of immunomodulatory role of a selected strain as probiotic. Marine biotechnology, 16(2), 181-192.
- Mirbakhsh, M., Akhavansepahy, A., Afsharnasab, M., Khanafari, A., & Razavi, M. R. (2013). Screening and evaluation of indigenous bacteria from the Persian Gulf as a probiotic and biocontrol agent against Vibrio harveyi in Litopenaeus vannamei post larvae. Iranian Journal of Fisheries Sciences, 12(4), 873-886.
- Najmi, N., Yahyavi, M., & Haghshenas, A. (2018). Effect of enriched rotifer (Brachionus plicatilis) with probiotic lactobacilli on growth, survival and resistance indicators of western white shrimp (Litopenaeus vannamei) larvae. Iranian Journal of Fisheries Sciences, 17(1), 11-20.
- Ngo, H. T., Nguyen, T. T. N., Nguyen, Q. M., Tran, A. V., Do, H. T. V., Nguyen, A. H., ... & Nguyen, A. T. V. (2016). Screening of pigmented Bacillus aquimaris SH 6 from the intestinal tracts of shrimp to develop a novel feed supplement for shrimp. Journal of applied microbiology, 121(5), 1357-1372.
- Nguyen, V. D., Pham, T. T., Nguyen, T. H. T., Nguyen, T. T. X., & Hoj, L. (2014). Screening of marine bacteria with bacteriocin-like activities and probiotic potential for ornate spiny lobster (Panulirus ornatus) juveniles. Fish & shellfish immunology, 40(1), 49-60
- Nimrat, S., Suksawat, S., Boonthai, T., & Vuthiphandchai, V. (2012). Potential Bacillus probiotics enhance bacterial numbers, water quality and growth during early development of white shrimp (Litopenaeus vannamei). Veterinary microbiology, 159(3-4), 443-450.
- Pane, L., Radin, L., Franconi, G., & Carli, A. (1996). The carotenoid pigments of a marine Bacillus firmus strain. Bollettino della Societa italiana di biologia sperimentale, 72(11-12), 303-308.
- Pinoargote, G., Flores, G., Cooper, K., & Ravishankar, S. (2018). Effects on survival and bacterial community composition of the aquaculture water and gastrointestinal tract of shrimp (Litopenaeus vannamei) exposed to probiotic treatments after an induced infection of acute hepatopancreatic necrosis disease. Aquaculture Research, 49(10), 3270-3288.
- Purivirojkul, W. (2013). Application of probiotic bacteria for controlling pathogenic bacteria in fairy shrimp Branchinella thailandensis culture. Turkish Journal of Fisheries and Aquatic Sciences, 13(1), 187-196.
- Purivirojkul, W., Maketon, M., & Areechon, N. (2005). Probiotic properties of Bacillus pumilus, Bacillus sphaericus and Bacillus subtilis in black tiger shrimp (Penaeus monodon Fabricius) culture. Agriculture and Natural Resources, 39(2), 262-273.
- Sánchez-Díaz, R., Molina-Garza, Z. J., Cruz-Suárez, L. E., Selvin, J., Kiran, G. S., Ibarra-Gámez, J. C., ... & Galaviz-Silva, L. (2019). Draft genome sequence of Pseudoalteromonas piscicida strain 36Y_RITHPW, a hypersaline seawater isolate from the south coast of Sonora, Mexico. Journal of global antimicrobial resistance, 16, 83-86.
- Satish, R. K., & Arul, V. (2009). Purification and characterization of phocaecin PI80: an anti-listerial bacteriocin produced by Streptococcus phocae PI80 Isolated from the gut of Peneaus indicus (Indian white shrimp). Journal of microbiology and biotechnology, 19(11), 1393-1400.
- Sha, Y., Liu, M., Wang, B., Jiang, K., Qi, C., & Wang, L. (2016). Bacterial population in intestines of Litopenaeus vannamei fed different probiotics or probiotic supernatant. J. Microbiol. Biotechnol, 26(10), 1736-1745.
- Sha, Y., Wang, B., Liu, M., Jiang, K., & Wang, L. (2016b). Interaction between Lactobacillus pentosus HC-2 and Vibrio parahaemolyticus E1 in Litopenaeus vannamei in vivo and in vitro. Aquaculture, 465, 117-123.
- Sha, Y., Wang, L., Liu, M., Jiang, K., Xin, F., & Wang, B. (2016c). Effects of lactic acid bacteria and the corresponding supernatant on the survival, growth performance, immune response and disease resistance of Litopenaeus vannamei. Aquaculture, 452, 28-36.
- Shakibazadeh, S., Saad, C. R., Christianus, A., Kamarudin, M. S., Sijam, K., & Sinaian, P. (2011). Assessment of possible human risk of probiotic application in shrimp farming. International Food Research Journal, 18(1).
- Shakibazadeh, S., Saad, C. R., Christianus, A., Kamarudin, M. S., Sijam, K., Shamsudin, M. N., & Neela, V. K. (2008). Evaluation of in vitro Vibrio static activity of Shewanella algae isolated from healthy Penaeus monodon. African Journal of Biotechnology, 7(21).
- Sivakumar, N., Sundararaman, M., & Selvakumar, G. (2012). Probiotic effect of Lactobacillus acidophilus against vibriosis in juvenile shrimp (Penaeus monodon). African Journal of Biotechnology, 11(91), 15811-15818.
- Sumon, M. S., Ahmmed, F., Khushi, S. S., Ahmmed, M. K., Rouf, M. A., Chisty, M. A. H., & Sarower, M. G. (2018). Growth performance, digestive enzyme activity and immune response of Macrobrachium rosenbergii fed with probiotic Clostridium butyricum incorporated diets. Journal of King Saud University-Science, 30(1), 21-28.
- Sundaram, M., Panigrahi, A., Ganesh, J., Rekha, P. N., Sivagnanam, S., Rajamanickam, S., & Gopal, C. (2017). Evaluation of different probiotic strains for growth performance and immunomodulation in Pacific white shrimp Penaeus vannamei Boone, 1931.
- Swain, S. M., Singh, C., & Arul, V. (2009). Inhibitory activity of probiotics Streptococcus phocae PI80 and Enterococcus faecium MC13 against vibriosis in shrimp Penaeus monodon. World Journal of Microbiology and Biotechnology, 25(4), 697-703.
- Tepaamorndech, S., Chantarasakha, K., Kingcha, Y., Chaiyapechara, S., Phromson, M., Sriariyanun, M., ... & Visessanguan, W. (2019). Effects of Bacillus aryabhattai TBRC8450 on vibriosis resistance and immune enhancement in Pacific white shrimp, Litopenaeus vannamei. Fish & shellfish immunology, 86, 4-13.
- Thompson, J., Gregory, S., Plummer, S., Shields, R. J., & Rowley, A. F. (2010). An in vitro and in vivo assessment of the potential of Vibrio spp. as probiotics for the Pacific White shrimp, Litopenaeus vannamei. Journal of applied microbiology, 109(4), 1177-1187.
- Van Hai, N., & Fotedar, R. (2009). Comparison of the effects of the prebiotics (Bio-Mos® and b-1,3-D-glucan) and the customised probiotics (Pseudomonas synxantha and P. aeruginosa) on the culture of juvenile western king prawns (Penaeus latisulcatus Kishinouye, 1896). Aquaculture, 289(3-4), 310-316.
- Van Hai, N., Fotedar, R., & Buller, N. (2007). Selection of probiotics by various inhibition test methods for use in the culture of western king prawns, Penaeus latisulcatus (Kishinouye). Aquaculture, 272(1-4), 231-239.
- Vieira, F. D. N., Jatobá, A., Mouriño, J. L. P., Vieira, E. A., Soares, M., Silva, B. C. D., ... & Vinatea, L. A. (2013). In vitro selection of bacteria with potential for use as probiotics in marine shrimp culture. Pesquisa Agropecuária Brasileira, 48(8), 998-1004.
- Vijayan, K. K., Singh, I. B., Jayaprakash, N. S., Alavandi, S. V., Pai, S. S., Preetha, R., ... & Santiago, T. C. (2006). A brackishwater isolate of Pseudomonas PS-102, a potential antagonistic bacterium against pathogenic vibrios in penaeid and non- penaeid rearing systems. Aquaculture, 251(2-4), 192-200.
- Villamil, L., Figueras, A., Planas, M., & Novoa, B. (2003). Control of Vibrio alginolyticus in Artemia culture by treatment with bacterial probiotics. Aquaculture, 219(1-4), 43-56.
- Wang, Y. B. (2007). Effect of probiotics on growth performance and digestive enzyme activity of the shrimp Penaeus vannamei. Aquaculture, 269(1-4), 259-264.
- Wang, Y. C., Hu, S. Y., Chiu, C. S., & Liu, C. H. (2019). Multiple-strain probiotics appear to be more effective in improving the growth performance and health status of white shrimp, Litopenaeus vannamei, than single probiotic strains. Fish & shellfish immunology, 84, 1050-1058.
- Wang, Y., & Gu, Q. (2010). Effect of probiotics on white shrimp (Penaeus vannamei) growth performance and immune response. Marine Biology Research, 6(3), 327-332.
- Wen, C. Q., Xue, M., Liang, H. F., Wu, Y., & Li, X. (2015). Beneficial effects of Ectothiorhodospira shaposhnikovii WF on larval cultivation of Litopenaeus vannamei. Beneficial microbes, 6(4), 525-533.
- Wen, C., Xue, M., Liang, H., & Zhou, S. (2014). Evaluating the potential of marine Bacteriovorax sp. DA5 as a biocontrol agent against vibriosis in Litopenaeus vannamei larvae. Veterinary microbiology, 173(1-2), 84-91.
- Xue, M., Liang, H., He, Y., & Wen, C. (2016). Characterization and in-vivo evaluation of potential probiotics of the bacterial flora within the water column of a healthy shrimp larviculture system. Chinese journal of oceanology and limnology, 34(3), 484-491.
- Xue, M., Wen, C., Liang, H., Ding, M., Wu, Y., & Li, X. (2016). In vivo evaluation of the effects of commercial Bacillus probiotics on survival and development of Litopenaeus vannamei larvae during the early hatchery period. Aquaculture research, 47(5), 1661-1669.
- You, J. L., Cao, L. X., Liu, G. F., Zhou, S. N., Tan, H. M., & Lin, Y. C. (2005). Isolation and characterization of actinomycetes antagonistic to pathogenic Vibrio spp. from nearshore marine sediments. World Journal of Microbiology and Biotechnology, 21(5), 679-682.
- Yuhana, M., & Zairin Jr, M. (2017). The nutritional value of Artemia sp. enriched with the probiotic Pseudoalteromonas piscicida and the prebiotic mannan-oligosaccharide. Aquaculture, Aquarium, Conservation & Legislation-International Journal of the Bioflux Society (AACL Bioflux), 10(1).
- Yuli, A., Ratu, S., Shaiyanne, F. (2018). Vannamei shrimp (Litopenaeus vannamei, Boone, 1931) performance with bacillus and lactobacillus probiotic formulation consortium in dry preparation. Research Journal of Chemistry and Environment, 22, 307-312
- Zadeh, S. S., Saad, C. R., Christianus, A., Kamarudin, M. S., Sijam, K., Shamsudin, M. N., & Neela, V. K. (2010). Assessment of growth condition for a candidate probiotic, Shewanella algae, isolated from digestive system of a healthy juvenile Penaeus monodon. Aquaculture international, 18(6), 1017-1026.
- Zhang, J., Lu, N., Tian, C., Li, Y., & Pan, L. (2017). Screening and identification of an efficient nitrogen-degrading strain and application of a potential analysis. Journal of Fishery Sciences of China, 24(4), 757-765. doi:10.3724/SP.J.1118.2017.16252
- Zheng, C. N., & Wang, W. (2017). Effects of Lactobacillus pentosus on the growth performance, digestive enzyme and disease resistance of white shrimp, Litopenaeus vannamei (Boone, 1931). Aquaculture research, 48(6), 2767-2777.
- Zhou, X. X., Wang, Y. B., & Li, W. F. (2009). Effect of probiotic on larvae shrimp (Penaeus vannamei) based on water quality, survival rate and digestive enzyme activities. Aquaculture, 287(3-4), 349-353.
