# Supplementary material for: Agavin induces beneficial microbes in the shrimp microbiota under farming conditions
Source: Sci Rep. 2022 Apr 16;12:6392. doi: 10.1038/s41598-022-10442-2 (PMC9013378; doi:10.1038/s41598-022-10442-2)
Supplement: Supplementary file 2 — Supplementary Information 2. [file 41598_2022_10442_MOESM2_ESM.zip › new_TABLE_s4.docx]

| **BD** | | | | | | |
| --- | --- | --- | --- | --- | --- | --- |
| **Sample** | **Organ** | **Reads per sample** | **Joined reads** | **Reads post-quality ( Q >20)** | **Assigned reads** | **Reads post-abundance filters** |
| C1H1 | H | 71376 | 52810 | 52764 | 36415 | 34678 |
| C1H2 | H | 50730 | 46282 | 46232 | 25370 | 24773 |
| C1H3 | H | 41254 | 34037 | 33988 | 26114 | 25355 |
| C1H4 | H | 315 | 131 | 131 | 72 | 49 |
| C1i1 | I | 73165 | 57204 | 57113 | 33056 | 30020 |
| C1i2 | I | 501 | 258 | 257 | 121 | 77 |
| C1i3 | I | 28607 | 22546 | 22513 | 12513 | 11213 |
| C1i4 | I | 48889 | 40847 | 40791 | 27316 | 24571 |
| C2H1 | H | 55038 | 49859 | 49817 | 28738 | 28135 |
| C2H2 | H | 545 | 293 | 292 | 118 | 80 |
| C2H3 | H | 15879 | 10926 | 10901 | 9263 | 8843 |
| C2H4 | H | 24499 | 13470 | 13441 | 8826 | 7521 |
| C2i1 | I | 10603 | 2962 | 2942 | 2372 | 2257 |
| C2i2 | I | 18973 | 9160 | 9131 | 6290 | 5761 |
| C2i3 | I | 62813 | 44036 | 43933 | 33386 | 31769 |
| C2i4 | I | 46678 | 32105 | 32033 | 19704 | 16893 |
| C3H1 | H | 15600 | 7636 | 7610 | 6499 | 6164 |
| C3H2 | H | 267 | 130 | 130 | 63 | 50 |
| C3H3 | H | 31153 | 26023 | 25985 | 19070 | 17592 |
| C3H4 | H | 5928 | 4277 | 4266 | 3348 | 3290 |
| C3i1 | I | 36023 | 25122 | 25064 | 18669 | 18240 |
| C3i2 | I | 104191 | 88348 | 88195 | 55391 | 50587 |
| C3i3 | I | 56619 | 37512 | 37417 | 27268 | 22594 |
| C3i4 | I | 81828 | 69012 | 68888 | 53359 | 51771 |

| **AG2** | | | | | | |
| --- | --- | --- | --- | --- | --- | --- |
| **Sample** | **Organ** | **Reads per sample** | **Reads after join** | **Reads post-quality ( Q > 20)** | **Assigned reads** | **Reads post-filters** |
| 2-1H1 | H | 136590 | 112823 | 112605 | 88345 | 84286 |
| 2-1H2 | H | 51792 | 34566 | 34472 | 23866 | 23503 |
| 2-1H3 | H | 39053 | 30673 | 30621 | 25878 | 25381 |
| 2-1H4 | H | 13616 | 4100 | 4083 | 2803 | 2460 |
| 2-1i1 | I | 62698 | 50572 | 50482 | 32995 | 30616 |
| 2-1i2 | I | 53280 | 44924 | 44834 | 24929 | 23438 |
| 2-1i3 | I | 64802 | 56390 | 56306 | 39065 | 37638 |
| 2-1i4 | I | 46150 | 32836 | 32775 | 23729 | 22199 |
| 2-2H1 | H | 1664 | 360 | 357 | 224 | 191 |
| 2-2H2 | H | 68691 | 58292 | 58186 | 47265 | 46516 |
| 2-2H3 | H | 75038 | 63060 | 62954 | 51822 | 49768 |
| 2-2H4 | H | 63704 | 56066 | 56006 | 35099 | 34081 |
| 2-2i1 | I | 75232 | 65594 | 65476 | 34044 | 31375 |
| 2-2i2 | I | 31142 | 20231 | 20191 | 16014 | 15622 |
| 2-2i3 | I | 52561 | 36399 | 36309 | 22451 | 21064 |
| 2-2i4 | I | 36617 | 24686 | 24625 | 17033 | 15312 |
| 2-3H1 | H | 55121 | 46756 | 46693 | 30658 | 29486 |
| 2-3H2 | H | 8821 | 4842 | 4835 | 3880 | 3114 |
| 2-3H3 | H | 126999 | 118119 | 117965 | 88572 | 81253 |
| 2-3H4 | H | 311 | 149 | 148 | 79 | 65 |
| 2-3i1 | I | 42024 | 29211 | 29136 | 16776 | 15630 |
| 2-3i2 | I | 59133 | 43812 | 43723 | 33099 | 32154 |
| 2-3i3 | I | 31762 | 24734 | 24689 | 15540 | 14882 |
| 2-3i4 | I | 61953 | 48905 | 48827 | 33844 | 31513 |

| **AG10** | | | | | | |
| --- | --- | --- | --- | --- | --- | --- |
| **Sample** | **Organ** | **Reads per sample** | **Reads after join** | **Reads post-quality ( Q > 20)** | **Assigned reads** | **Reads post-filters** |
| 10-1H1 | H | 32904 | 27082 | 27034 | 19249 | 17873 |
| 10-1H2 | H | 64531 | 39652 | 39557 | 31565 | 29665 |
| 10-1H3 | H | 49299 | 32786 | 32723 | 23350 | 20979 |
| 10-1H4 | H | 92734 | 63216 | 63103 | 51696 | 49726 |
| 10-1i1 | I | 77137 | 58183 | 58075 | 35350 | 31475 |
| 10-1i2 | I | 36225 | 12358 | 12298 | 8427 | 6878 |
| 10-1i3 | I | 54491 | 40839 | 40770 | 28275 | 25814 |
| 10-1i4 | I | 23354 | 5095 | 5069 | 3520 | 2826 |
| 10-2H1 | H | 23710 | 16295 | 16255 | 13111 | 12663 |
| 10-2H2 | H | 30107 | 17833 | 17791 | 13264 | 11861 |
| 10-2H3 | H | 51767 | 39526 | 39470 | 29197 | 26467 |
| 10-2H4 | H | 423 | 221 | 220 | 123 | 87 |
| 10-2i1 | I | 27384 | 23708 | 23663 | 18309 | 17686 |
| 10-2i2 | I | 48291 | 39543 | 39480 | 26714 | 25788 |
| 10-2i3 | I | 75281 | 63173 | 63094 | 45062 | 41630 |
| 10-2i4 | I | 93460 | 80324 | 80207 | 52888 | 44879 |
| 10-3H1 | H | 67905 | 48027 | 47950 | 39463 | 37323 |
| 10-3H2 | H | 17547 | 12233 | 12207 | 9902 | 9147 |
| 10-3H3 | H | 76429 | 59539 | 59461 | 49855 | 46211 |
| 10-3i1 | I | 49641 | 37971 | 37917 | 23518 | 21181 |
| 10-3i2 | I | 68695 | 56678 | 56564 | 41330 | 38785 |
| 10-3i3 | I | 47723 | 36354 | 36309 | 25338 | 23768 |
| 10-3i4 | I | 109659 | 93490 | 93348 | 62488 | 56569 |

Table S4. Total reads before and after quality and abundance filters for each sample. H: Hepatopancreas. I: Intestine. The legends in the labels of the sample name corresponds as follows, ie: for sample C1H1, C, = Basal diet, 1 = cage 1, H = Hepatopancreas, 1 = Shrimp 1. For sample 2-1H1, 2 = Agavine 2 % diet, 1 = cage 1, H = Hepatopancreas, 1 = Shrimp 1. For sample 10-1H1, 10 = Agavine 10 % diet, 1 = cage 1, H = Hepatopancreas, 1 = Shrimp 1
