# Supplementary material for: Agavin induces beneficial microbes in the shrimp microbiota under farming conditions
Source: Sci Rep. 2022 Apr 16;12:6392. doi: 10.1038/s41598-022-10442-2 (PMC9013378; doi:10.1038/s41598-022-10442-2)
Supplement: Supplementary file 2 — Supplementary Information 2. [file 41598_2022_10442_MOESM2_ESM.zip › new_TABLE_s5.docx]

| *Bacillus amyloliquefaciens* | *Bifidobacterium bifidum* | *Pediococcus acidilactici MA18/5M* |
| --- | --- | --- |
| *Bacillus cereus* | *Bifidobacterium thermophilum* | *Pseudoalteromonas piscicida* |
| *Bacillus circulans* | *Clostridium butyricum* | *Pseudomonas aeruginosa* |
| *Bacillus coagulans* | *Enterobacter hormaechei subsp. hormaechei* | *Pseudomonas putida* |
| *Bacillus firmus* | *Enterococcus faecium* | *Pseudomonas putida ** |
| *Bacillus firmus ** | *Lactobacillus delbrueckii* | *Pseudomonas putida B6-2* |
| *Bacillus licheniformis* | *Lactobacillus fermentum* | *Pseudoalteromonas ruthenica CP76* |
| *Bacillus megaterium WSH-002* | *Lactobacillus pentosus* | *Pseudomonas synxantha* |
| *Bacillus pumilus* | *Lactobacillus plantarum* | *Rhodopseudomonas palustris* |
| *Bacillus subtilis* | *Lactobacillus reuteri* | *Streptococcus thermophilus TH1435* |
| *Bacillus subtilis subsp. subtilis str. 168* | *Lactococcus lactis subsp. lactis* | *Vibrio alginolyticus* |
| *Bacillus tequilensis* | *Lysinibacillus fusiformis* | *Vibrio fluvialis* |
| *Bacillus thuringiensis* | *Paenibacillus polymyxa* | *Vibrio hepatarius* |
| *Bdellovibrio bacteriovorus* | *Pediococcus acidilactici* | *Vibrio mediterranei* |

Table S5. List of probiotic species found in the shrimp samples. The * in duplicate species indicates that these were found under different taxonomic levels in the Silva 132 database.
